# A *p*-Coumaroyl-CoA Biosensor for Dynamic Regulation of Naringenin Biosynthesis in *Saccharomyces cerevisiae*

**DOI:** 10.1021/acssynbio.2c00111

**Published:** 2022-09-22

**Authors:** Dany Liu, Maria Sole Sica, Jiwei Mao, Lucy Fang-I Chao, Verena Siewers

**Affiliations:** Department of Biology and Biological Engineering, Chalmers University of Technology, SE-412 96 Gothenburg, Sweden

**Keywords:** yeast, flavonoids, transcriptional regulation, transcription repressor, dynamic pathway control

## Abstract

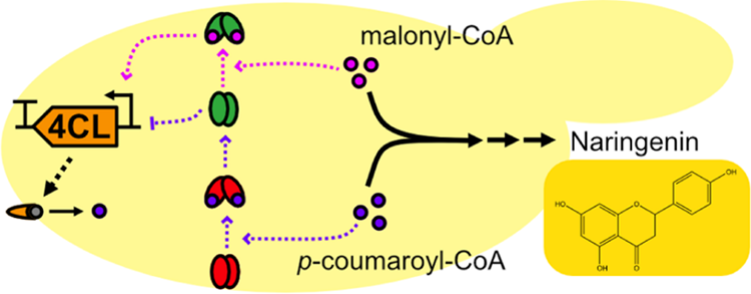

In vivo biosensors that can convert metabolite concentrations into measurable output signals are valuable tools for high-throughput screening and dynamic pathway control in the field of metabolic engineering. Here, we present a novel biosensor in *Saccharomyces cerevisiae* that is responsive to *p*-coumaroyl-CoA, a central precursor of many flavonoids. The sensor is based on the transcriptional repressor CouR from *Rhodopseudomonas palustris* and was applied in combination with a previously developed malonyl-CoA biosensor for dual regulation of *p*-coumaroyl-CoA synthesis within the naringenin production pathway. Using this approach, we obtained a naringenin titer of 47.3 mg/L upon external precursor feeding, representing a 15-fold increase over the nonregulated system.

## Introduction

Flavonoids are a class of phytochemicals, which exhibit biological activities that are beneficial for human health.^1^ Several studies have shown that flavonoids have positive effects against certain diseases, such as cardiovascular diseases,^2,3^ diabetes,^4^ and cancer.^5−7^ For this reason, interest in the production and commercialization of flavonoids has increased significantly over the last two decades. One important flavonoid is the flavanone naringenin, as it serves as a precursor of many other flavonoids, including flavones, flavonols, isoflavonoids, and so forth.^8^

Current flavonoid production relies largely on solvent extraction from plants and suffers from low efficiencies and high costs due to long extraction times, low extraction selectivity, the need for large amounts of high-purity solvents, and possible degradation of the extracted compounds.^9^ Chemical synthesis, on the other hand, is impeded by the structural complexity of some flavonoids and the requirement for harsh operating conditions.^10^ The use of microbial cell factories thus presents a promising alternative, bypassing many of the challenges connected to extraction and organic synthesis.^11,12^ Baker’s yeast *Saccharomyces cerevisiae* is a commonly used model organism for metabolic engineering purposes and has been explored extensively for the production of flavonoids, including naringenin. It is easy to manipulate genetically, has a high tolerance toward industrial fermentation conditions, and possesses eukaryotic organelles and membranes necessary for the functional expression of certain plant enzymes.^13^ Many studies focusing on precursor overproduction,^14,15^ flavonoid assembly,^16,17^ and downstream functionalization^18,19^ have been conducted. To date, the highest naringenin titers achieved from *p*-coumaric acid and glucose in bioreactor fermentations are 1.21 g/L in *S. cerevisiae* and 898 mg/L in *Yarrowia lipolytica,* respectively.^17,20^ Nevertheless, titers, rates, and yields must be further improved before industrial applications will be possible.

**Figure 1:**
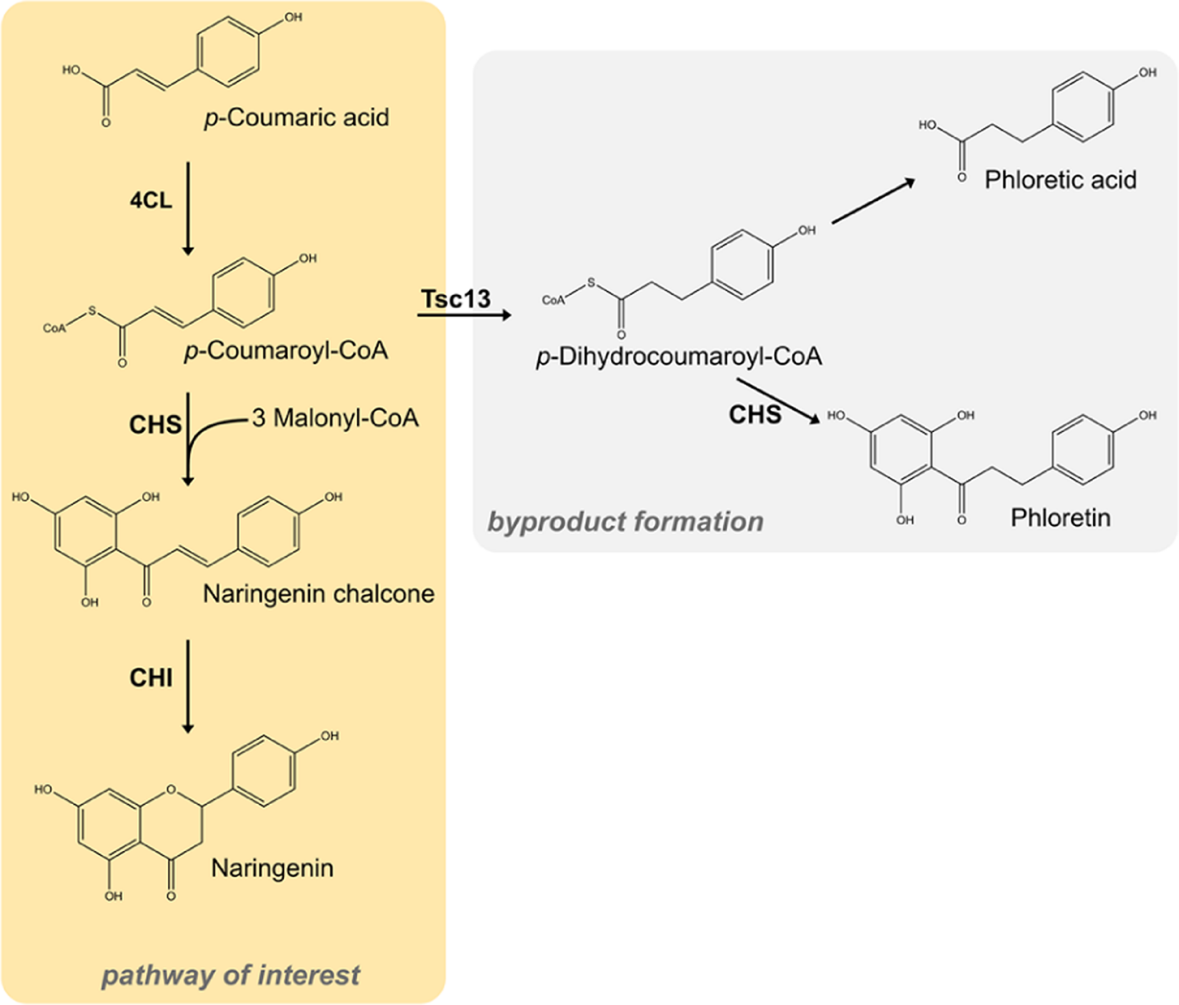
**Naringenin biosynthetic pathway and potential byproduct formation in*S. cerevisiae***. The conversion of *p*-coumaric acid to naringenin involves three enzymes, 4-coumarate:CoA ligase (4CL), chalcone synthase (CHS), and chalcone isomerase (CHI). The unspecific activity of an endogenous enoyl reductase (Tsc13) leads to reduction of *p*-coumaroyl-CoA to *p*-dihydrocoumaroyl-CoA, which results in the formation of two byproducts, phloretic acid and phloretin.

The biosynthesis of naringenin proceeds via l-phenylalanine and l-tyrosine, which are converted to *p*-coumaric acid in the shikimate pathway. *p*-Coumaric acid is activated to *p*-coumaroyl-CoA by a 4-coumarate:CoA ligase (4CL), which is then converted to naringenin chalcone by successive condensations with three malonyl-CoA moieties. This reaction is catalyzed by a chalcone synthase (CHS). The final step is the cyclization of naringenin chalcone to naringenin by a chalcone isomerase (CHI).^8^ Potential byproducts during naringenin synthesis include phloretic acid and phloretin (Figure 1).

A major challenge in the microbial production of natural products such as flavonoids is that long heterologous pathways can create a metabolic burden on the cell factory, subsequently limiting its productivity.^21,22^ Dividing the labor over multiple strains in a microbial consortium would minimize the amount of genetic engineering required of and cell stress put on each strain. In addition, one could leverage the unique characteristics of different species. It would also allow optimization of separate modules of a complex pathway in parallel rather than in sequence. Several studies have reported stable same- or different-species consortia for improved production of natural products.^23−26^

Traditionally, metabolic engineering of microbial cell factories relies on static (over) expression of pathway enzymes. This can create flux imbalances which often lead to suboptimal fermentation results. Therefore, dynamic pathway control to optimally allocate carbon and energy resources between cell growth and production has been explored progressively in recent times.^27−29^ This approach is particularly useful for microbial consortia, due to the additional variability in metabolite levels that arises from coculturing multiple organisms. It can also be beneficial in heterologous pathways, where non-native enzymes or metabolites may prove to be toxic to the host organism.^30−32^ Although several successful examples of dynamic pathway control exist in *Escherichia coli*,^29,30,32−37^ implementations in yeast are still rare.^38^ This is partly due to the lack of metabolite biosensors which are necessary for the development of such genetic circuits.^27,39^

For naringenin production, the CoA thioesters *p*-coumaroyl-CoA and malonyl-CoA serve as key intermediates. A FapR transcription factor-based fluorescence biosensor and an enzyme-based colorimetric biosensor have previously been established for malonyl-CoA.^38,40,41^*p*-Coumaric acid can also be detected using a PadR transcription factor-based system.^42,43^ However, a *p*-coumaroyl-CoA biosensor does not exist. Such a sensor would be useful for optimizing 4CL or CHS activity by high-throughput screening and could also be used to regulate *p*-coumaroyl-CoA production within the naringenin biosynthetic pathway.

With this in mind, we constructed a yeast strain that can be employed in a consortium with a *p*-coumarate and a malonate overproducer to efficiently assemble these two precursors to form naringenin. We further developed a *p*-coumaroyl-CoA-responsive transcriptional repressor-based biosensor and repurposed it for dynamic regulation of the naringenin pathway by transcriptional modulation of *4CL* in response to *p*-coumaroyl-CoA and malonyl-CoA availability.

## Results

### Designing a *p*-Coumaroyl-CoA Sensor in *S. cerevisiae*

The bacterial transcription repressor CouR from the MarR family of transcription factors has been shown to negatively regulate a set of *cou* genes, which are responsible for *p*-coumarate catabolism in *Rhodopseudomonas palustris* and *Rhodococcus jostii*.^44−47^ Furthermore, it was shown that CouR specifically binds to *p*-coumaroyl-CoA and not to *p*-coumarate, coenzyme A, or any of the *p*-coumarate degradation products.^46,47^ The crystal structures and DNA binding sequences of both *R. palustris* and *R. jostii* CouR (hereafter RpCouR and RjCouR) have been characterized previously.^44,47^ CouR forms a homodimer and binds to a palindromic operator sequence through a winged helix-turn-helix motif. Each protomer binds one ligand molecule. The phenolic moieties of the *p*-coumaroyl-CoA ligand occupy hydrophobic pockets of the protein, while the CoA moieties are predicted to abrogate CouR-DNA interactions through steric hindrance and electrostatic repulsion. Despite having similar names, RjCouR and RpCouR only share 36% sequence identity. Their DNA binding sites also differ in length and sequence.^44^ We thus selected both RjCouR and RpCouR as sensing components of our *p*-coumaroyl-CoA biosensor.

In the case of transcriptional repressor-based biosensors, the binding of the repressor to the operator sequence represses reporter expression in the absence of the ligand. With increasing ligand concentration in the cell, the ligand will attenuate repressor–operator interactions, allowing the reporter to be expressed. The correlation between the reporter signal and ligand concentration gives us a qualitative indication of the amount of metabolite being produced.

To enable CouR-DNA binding, the CouR-specific operator sequence must be inserted into the promoter that controls reporter expression. The positioning of the operator site(s) within the promoter is crucial for proper DNA binding and subsequent transcriptional repression. At the same time, the insertion of the operator site should not severely disrupt the native promoter activity to provide a sufficient dynamic range, that is, a large ratio between maximal fluorescence in the presence of the ligand and baseline fluorescence in its absence.

**Figure 2:**
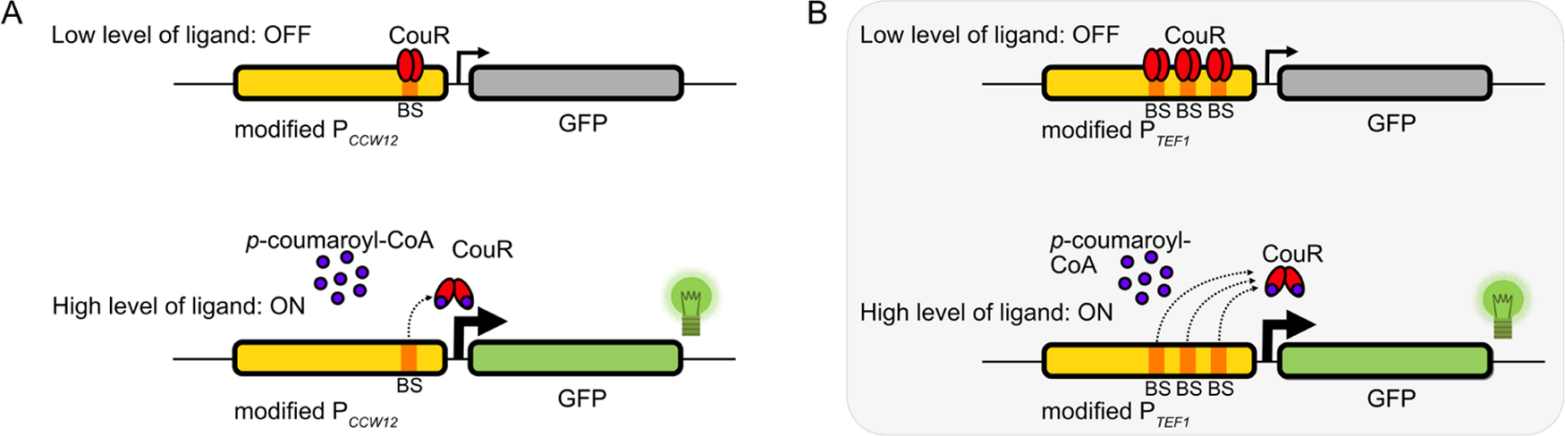
***p*-Coumaroyl-CoA biosensor based on a transcriptional repressor CouR, derived from*R. palustris*and*R. jostii*.** Two modified promoters, **(A)** P_*CCW12*_ with one CouR DNA binding site (BS) and **(B)** P_*TEF1*_ with three CouR DNA binding sites, were tested. At low ligand concentrations, CouR represses GFP expression (OFF). At high ligand concentrations, the ligand binds CouR and releases it from its DNA binding site, leading to increased GFP expression (ON).

Although there are many unknowns and no general guidelines regarding the design of sensor systems and operator positioning,^48^ a number of biosensors have been implemented successfully in yeast. One well-studied system is the FapR-based biosensor. FapR is a transcriptional repressor originating from *Bacillus subtilis* that recognizes malonyl-CoA as its ligand. It has previously been employed in *S. cerevisiae* by our laboratory,^38^ where three FapR DNA binding sites were inserted into the strong constitutive *TEF1* promoter (P_*TEF1*_) for control of GFP expression, yielding a sevenfold increase in fluorescence when comparing the presence and absence of FapR. Additionally, we identified several operator locations in different yeast native promoters that are potentially applicable not only to FapR but also to other transcription factors.^49^ The highest apparent dynamic range, that is, the largest difference between the complete absence and presence of the repressor, was achieved using the *CCW12* promoter (P_*CCW12*_) with a single DNA binding site inserted downstream of the P_*CCW1*2_ TATA box (P_*CCW12*_BS_2_). Based on these previous publications, we tested the proposed binding site locations (three in P_*TEF1*_ and one in P_*CCW12*_) in the CouR biosensor system (Figure 2). Both CouR variants, RjCouR and RpCouR, were assessed. The biosensor constructs were designed to contain the GFP reporter and the CouR repressor cassettes on a single CEN-ARS plasmid to minimize discrepancies caused by variations in the plasmid copy number.

### Biosensor Characterization

**Figure 3:**
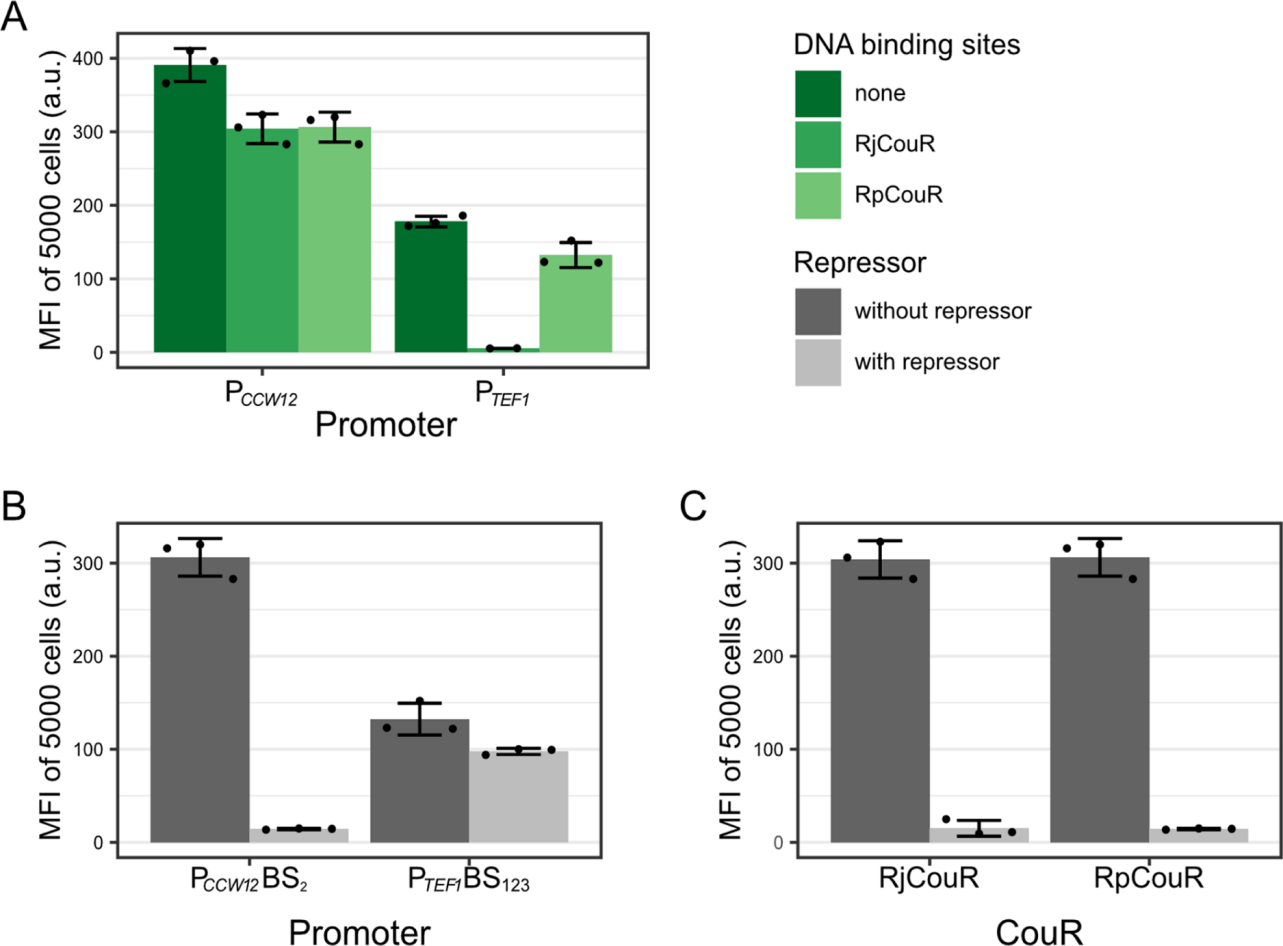
**Characterization of the CouR biosensor before *p*-coumaroyl-CoA induction. (A)** Effect of binding site insertions on P_*CCW12*_ and P_*TEF1*_ activity. **(B)** Comparison of fluorescence intensities of the modified P_*CCW12*_ and P_*TEF1*_ promoter in the presence or absence of RpCouR (pDL031 and pDL033). **(C)** Comparison of fluorescence intensities of RjCouR and RpCouR transcriptional repression in combination with the modified P*_CCW12_* promoter (pDL030 and pDL031). Strains were cultured in 250 μL of Delft medium in a 96-well plate, and the fluorescence intensity of 5000 cells was measured after 7 h of cultivation at 30 °C, 250 rpm shaking. The experiment was carried out in biological triplicates. Error bars represent standard deviation.

To determine whether the insertion of CouR DNA binding sites would impact native promoter activity, the fluorescence intensities of the wild-type P_*TEF1*_ and P_*CCW12*_ promoters regulating GFP expression were compared to those of the modified promoters with integrated operator sequences. As shown in Figure 3A, the insertion of one RpCouR or RjCouR binding site lowered the P_*CCW12*_-GFP median fluorescence intensity (MFI) by ca. 22%. The P_*TEF1*_ promoter modified with three RpCouR binding sites was still functional despite a reduction in basal promoter activity by 26%. Contrarily, the insertion of three RjCouR binding sites in P_*TEF1*_ diminished GFP expression completely.

We thus moved forward with three promoter versions, P_*CCW12*_BS_2,RjCouR_, P_*CCW12*_BS_2,RpCouR_, and P_*TEF1*_BS_123,RpCouR_ (full sequences in Supporting Information, Table S1) and measured their respective maximum dynamic ranges by comparing GFP expression changes in the presence and absence of the repressor. When comparing the P_*TEF1*_ promoter with three RpCouR-DNA binding sites (P_*TEF1*_BS_123,RpCouR_) and the P_*CCW12*_ promoter with one RpCouR-DNA binding site (P_*CCW12*_BS_2,RpCouR_), fold changes of 1.3x and 21.4x were observed (Figure 3B). The maximum dynamic range of RpCouR and RjCouR in combination with the respective P_*CCW12*_BS_2_ promoter (P_*CCW12*_BS_2,RpCouR_ and P_*CCW12*_BS_2,RjCouR_) were similar, with fold changes of 21.4x and 20.1x (Figure 3C). Since the modified P_*CCW12*_ exhibited considerably higher maximum dynamic ranges compared to the modified P_*TEF1*_ for both CouR variants, these two constructs were further characterized regarding their derepression performance.

The next step was to investigate the biosensor dynamics in response to the ligand. Since *p*-coumaroyl-CoA itself is not commercially available, we attempted feeding *p*-coumarate to a *4CL* expressing strain, yFlav13, in order to produce the ligand in vivo. However, the supplementation of *p*-coumarate posed a severe growth impediment to this strain (Supporting Information, Figure S1). Even delayed induction after an initial growth phase of 18 h in the absence of *p*-coumarate could not prevent growth arrest (data not shown). This indicated that the accumulation of *p*-coumaroyl-CoA without any downstream consumption was toxic to yeast. Similar growth-inhibiting effects have been observed in an *Acinetobacter* species, *E. coli,* and *Pseudomonas putida*.^50,51^ Consequently, it was not feasible to assess the biosensor response to *p*-coumaroyl-CoA using this strain.

**Figure 4:**
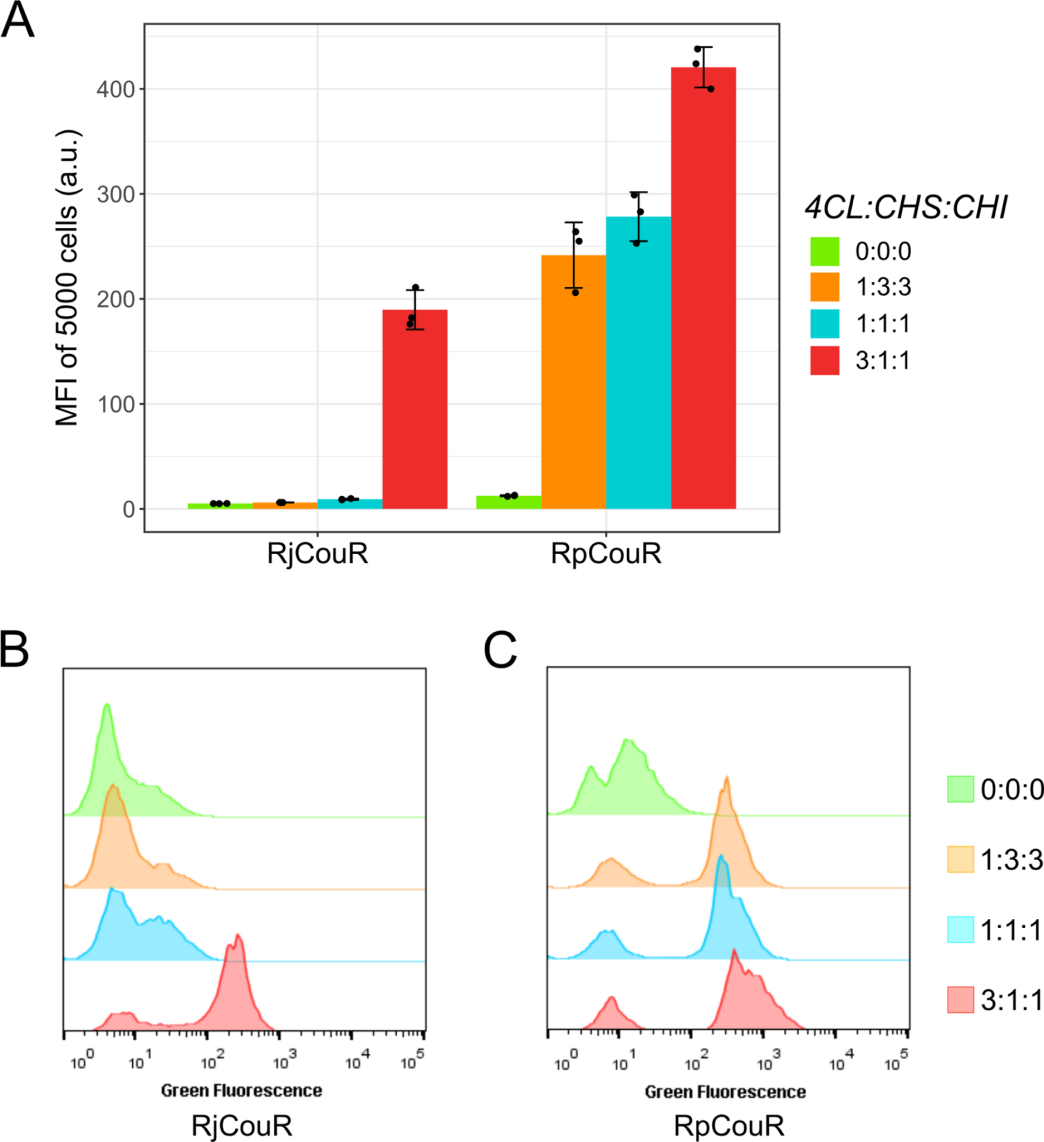
**Biosensor response to *p*-coumaroyl-CoA in naringenin strains QL11, NAG1-3, NAG10, and NAG3-1 with different copy numbers of *4CL*, *CHS*, and *CHI*. (A)** MFI values for each strain carrying the RjCouR- and RpCouR-based biosensors. Strains were cultivated in 3 mL of Delft medium, and samples were taken after 18 h of growth at 30 °C, 200 rpm shaking. Fluorescence was measured using flow cytometry. The experiment was carried out in triplicates. Error bars represent standard deviation. **(B)** Green fluorescence histograms of individual clones carrying the RjCouR biosensor. **(C)** Green fluorescence histograms of individual clones carrying the RpCouR biosensor. Fluorescence intensities of 5000 cells were acquired for each sample.

Instead, naringenin production strains with different copy numbers of the three pathway genes *4CL*, *CHS*, and *CHI* were used to characterize the GFP derepression (Figure 4). Strains with *4CL*:*CHS*:*CHI* copy number ratios of 0:0:0 (negative control, QL11^15^), 1:1:1 (NAG10), 1:3:3 (NAG1-3), and 3:1:1 (NAG3-1) were employed (Figure 1). It was expected that a copy number ratio of 1:3:3 would generate the lowest fluorescence intensities as three copies of *CHS* and *CHI* would effectively pull flux away from *p*-coumaroyl-CoA toward naringenin. Similarly, we suspected a ratio of 3:1:1 to induce the highest GFP expression, as three copies of *4CL* would bring about the highest level of *p*-coumaroyl-CoA accumulation. Using the RjCouR-based biosensor, strains 1:3:3 and 1:1:1 presented near base-level fluorescence. Only for strain 3:1:1, a notable increase in MFI was observed (Figure 4A,B). The RpCouR biosensor, on the other hand, exhibited a more gradual increase in MFI in the order 1:3:3 < 1:1:1 < 3:1:1 (Figure 4A,C) which was consistent with our predictions. The metabolite concentrations in all strains were quantified (Supporting Information, Figure S6), except for *p*-coumaroyl-CoA, which could not be detected by HPLC. Moreover, the control strain (0:0:0), which only produced *p*-coumaric acid and no *p*-coumaroyl-CoA,^15^ showed the lowest MFI values, affirming the high specificity of both CouR variants toward the CoA thioester compared to the nonesterified aromatic acid at intracellular levels. The histograms in Figure 4B,C display two separated cell populations, indicating a binary behavior of the biosensor. The cells are split into a fluorescent (induced) and a non-/low-fluorescent (noninduced) population. Therefore, the increase in MFI with increasing *p*-coumaroyl-CoA levels is due to both an increase in the ratio of induced cells over noninduced cells and a shift toward higher fluorescence intensities (see overlaid histograms of two independent experiments in Supporting Information, Figure S4). The population of nonfluorescent cells may at least partially result from plasmid instability as CouR expression was shown to reduce growth rates in yeast and lower percentage of plasmid-containing cells (Supporting Information, Figures S2 and S3). This plasmid loss was more pronounced in the case of RjCouR than RpCouR. The concentration threshold and operational range of the sensor were not quantified as *p*-coumaroyl-CoA could not be detected using standard analytical methods.

We also tested the response of RjCouR and RpCouR to other compounds in the naringenin biosynthetic pathway, namely, *p*-coumaric acid, naringenin, phloretin, and phloretic acid, by the addition of these to the medium at the highest soluble concentrations (Supporting Information, Figure S5). Both RjCouR and RpCouR showed no induction by naringenin, phloretin, and phloretic acid. However, RjCouR showed a slight response to *p*-coumaric acid when added at a concentration of 1 g/L.

### Biosensor Application for Dynamic Pathway Regulation

We next constructed a strain suitable for coculture production of naringenin from precursors *p*-coumarate and malonate. While *p*-coumarate is readily taken up by yeast cells, malonate uptake and activation must be engineered by introducing a malonate transporter and malonyl-CoA synthetase.^52^ As malonyl-CoA availability has been reported as a bottleneck in flavonoid biosynthesis,^53−55^ we anticipated the introduction of a malonate assimilation pathway via a malonate transporter (SpMae1 from *Schizosaccharomyces pombe*(52)) and a malonyl-CoA synthetase (RtMatB from *Rhizobium leguminosarumbv. trifolii*(52,53)) to improve precursor supply for naringenin production. Naturally, this heterologous pathway required supplementation with malonate.

**Figure 5:**
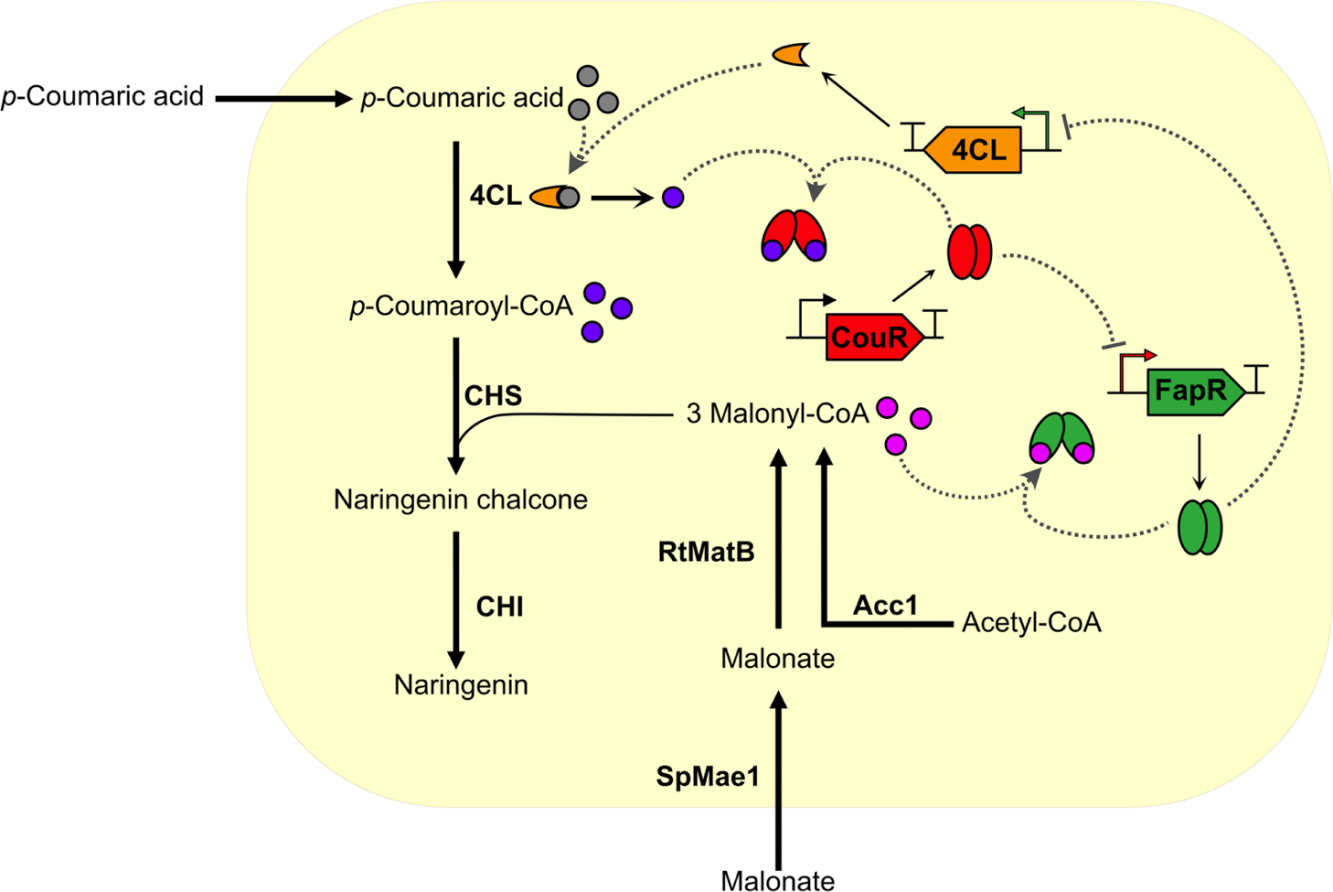
**Dynamic regulation of *4CL* in the naringenin synthetic pathway.** The strain contains one copy of *4CL*, four copies of *CHS*, and four copies of *CHI* for naringenin production from supplemented *p*-coumarate and malonate. Malonyl-CoA is produced endogenously by Acc1-catalyzed carboxylation of acetyl-CoA. A malonate transporter, SpMae1, and malonyl-CoA synthetase, RtMatB, were introduced for malonate assimilation to further increase the malonyl-CoA supply. The transcriptional repressor FapR was utilized to regulate *4CL* expression in response to intracellular malonyl-CoA levels, while CouR was employed for indirect control of *4CL* expression through FapR, in feedback to *p*-coumaroyl-CoA concentrations in the cell.

Due to the *p*-coumaroyl-CoA toxicity observed in previous experiments, we devised a dynamic regulatory circuit to modulate *p*-coumaroyl-CoA production in real time, based on the amount of *p*-coumaroyl-CoA and malonyl-CoA present in the cell (Figure 5). We first constructed a strain (yMS04) possessing one copy of *4CL* and four copies of *CHS* and *CHI* each, based on strain yFlav06 expressing genes *SpMae1* and *RtMatB*. To alleviate the toxicity that *p*-coumaroyl-CoA accumulation imposed on the cells, the aforementioned FapR transcriptional repressor was applied to regulate *4CL* expression depending on the amount of intracellular malonyl-CoA (strain yMS05). At lower malonyl-CoA concentrations, FapR would primarily bind to its DNA recognition site, thus downregulating *4CL* expression and limiting *p*-coumaroyl-CoA synthesis. At higher malonyl-CoA levels, the ligand would increasingly disrupt FapR-DNA interactions, leading to more *4CL* to be expressed. In a second step (strain yMS06), RpCouR was employed to regulate *4CL* indirectly through FapR in response to *p*-coumaroyl-CoA, creating a secondary feedback loop. RpCouR was chosen over RjCouR due to its stronger response to *p*-coumaroyl-CoA and its higher specificity. At low *p*-coumaroyl-CoA concentrations, CouR would repress FapR expression, which would result in *4CL* derepression and increased *p*-coumaroyl-CoA production. Elevated *p*-coumaroyl-CoA concentrations would then induce FapR transcription through CouR, resulting in *4CL* downregulation and decreased *p*-coumaroyl-CoA synthesis.

**Figure 6:**
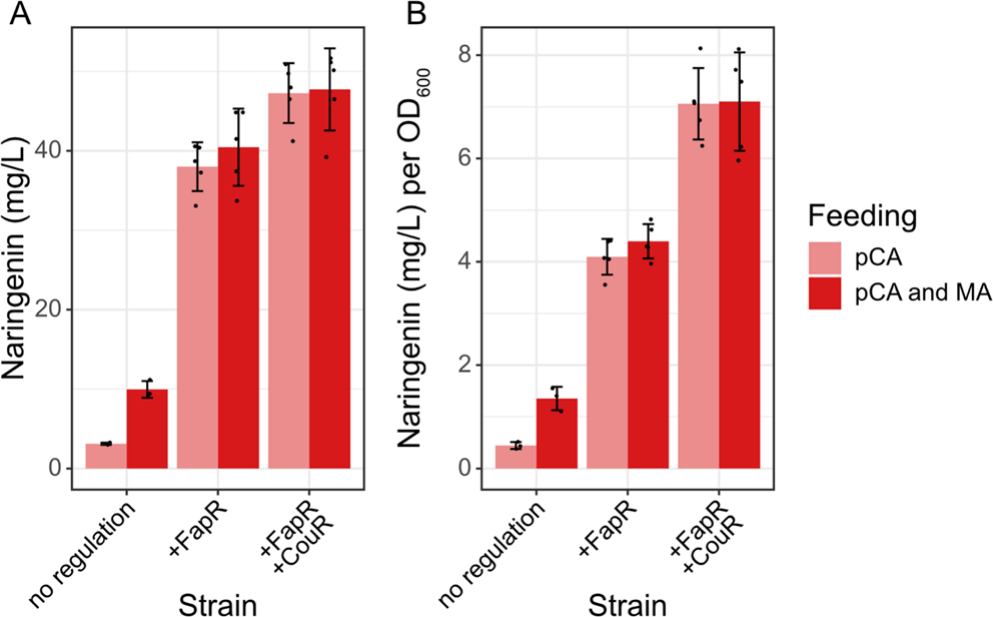
**Naringenin production in the nonregulated (yMS04), FapR-regulated (yMS05), and FapR-/CouR-regulated (yMS06) strains after 3 d of cultivation at 30 °C, 220 rpm. (A)** Total naringenin titers. **(B)** Naringenin titers normalized to biomass (OD_600_). Strains were grown in 20 mL of Delft medium for 4 d, and 0.75 mg of *p*-coumarate (pCA) in absolute ethanol (18.8 μL of 40 mg/L stock solution) and 3 mg of malonate (MA) in water (28.8 μL of 104 g/L stock solution) were added every 12 h. Samples were taken for HPLC analysis every 24 h. As metabolite concentrations became stagnant after 3 d of cultivation, the 72 h time point was used for strain comparison. Three and five replicates were assessed for the nonregulated and regulated strains, respectively. Error bars represent standard deviation.

The three naringenin production strains, nonregulated, FapR-regulated, and FapR-/CouR-regulated, were evaluated through the addition of 0.75 mg of *p*-coumarate (ca. 37.5 mg/L) and 3 mg of malonate (ca. 150 mg/L) every 12 h over 4 d of cultivation. Since metabolite concentrations became stagnant after 3 d of cultivation, the 72 h time point was used for comparison (Supporting Information, Figure S8). As seen in Figure 6A, the incorporation of FapR to regulate *4CL* expression through malonyl-CoA availability led to significant improvements in naringenin titers from 3.12 ± 0.13 to 38.0 ± 3.07 mg/L when comparing samples without malonate feeding. The integration of CouR further elevated titers to 47.3 ± 3.77 mg/L. Although the inclusion of CouR in the pathway control system only accounted for a 24% improvement in total naringenin titers, the naringenin produced per biomass was actually increased by 70% compared to sole FapR regulation (Figure 6B). It is also worth noting that while the FapR-/CouR-regulated strain was able to (almost) fully deplete supplemented *p*-coumaric acid, both the nonregulated and FapR-regulated strains showed residual *p*-coumaric acid after 3 d of cultivation (Supporting Information, Figure S7).

Two byproducts, phloretin and phloretic acid, arise from the naringenin pathway in *S. cerevisiae* (Figure 1). These two compounds are derived from the endogenous conversion of *p*-coumaroyl-CoA to its hydrogenation product *p*-dihydrocoumaroyl-CoA by an enoyl reductase Tsc13.^56,57^ The formation of phloretic acid was speculated to occur spontaneously or through native enzymes,^57^ whereas phloretin is a product of unspecific chalcone synthase activity. Besides higher naringenin titers, the regulated naringenin strains demonstrated an improved naringenin/phloretin and naringenin/phloretic acid ratio (Supporting Information, Figure S9).

The supplementation with malonate had a positive effect on the nonregulated strain, boosting naringenin titers threefold to 9.95 ± 1.05 mg/L, while also improving the naringenin/byproduct ratio considerably (Supporting Information, Figure S9). However, the addition of malonate did not have a significant impact on naringenin production or byproduct formation in either of the regulated strains.

## Discussion

We established a *p*-coumaroyl-CoA-responsive transcription factor-based biosensor with a high maximum dynamic range that can sense the ligand at physiologically relevant concentrations. Due to difficulties regarding chromatographic or mass spectrometric quantification of intracellular CoA thioesters and the inaccessibility of *p*-coumaroyl-CoA as an analytical standard, it was not possible to characterize the biosensor’s response dynamics and its absolute operational range by direct ligand feeding. Nonetheless, the sensor displayed gradual increases in fluorescence intensity when applied in different naringenin production strains, suggesting its suitability for metabolic engineering applications. However, one should be aware of the growth-inhibiting effect of CouR and the biosensor’s predominantly binary behavior. This may be due to biosensor plasmid loss and could potentially be avoided by genomic integration of the CouR and GFP expression cassettes. The fact that the biosensor is based on a prokaryotic transcriptional repressor means that it is unlikely to interact with native yeast metabolism. The orthogonality of the system also eliminates the reliance on endogenous transcription factors or RNA polymerase recruitment. The chosen DNA binding sites have been applied in a previous FapR biosensor setup,^49^ where the authors suggested universality of the identified operator locations. The successful transfer from FapR to CouR in this study further supports this hypothesis. The modified P_*CCW12*_ promoter emerged as superior to the modified P_*TEF1*_ promoter, consistent with observations made in the FapR biosensor.^49^ Surprisingly, the insertion of three RjCouR binding sites fully abolished P_*TEF1*_ activity, which was not the case when inserting three RpCouR binding sites in the same positions. The cause for damaged promoter activity has not been identified as differences in the RjCouR and RpCouR binding sequence length (29 and 31 bp) and GC content (31 and 32%) are minuscule.

The discrepancy in derepression behavior between the RjCouR- and the RpCouR-based biosensor may be attributed to a difference in expression levels or ligand affinity, although the reported dissociation constants of RjCouR and RpCouR (*K*_D,RjCouR_ = 11 ± 1 μM^47^ and *K*_D,RpCouR_ = 68 ± 8 μM^44^) would imply the opposite behavior. The derepression could also be influenced by the disparate growth rates of RjCouR and RpCouR strains. The stronger growth-inhibiting effect of RjCouR may impact both the metabolic activity and GFP expression of the cells. The reason for the growth retarding effect of CouR on yeast cells was not investigated but could be related to undesired interactions, either between CouR and the yeast genome or between CouR and other CoA thioesters or cellular components. In future studies, one might consider utilizing the biosensor to, for example, improve 4CL and CHS enzyme activity. As a *p*-coumaric acid-responsive biosensor already exists,^42,43^ one could also envision a dual screening approach with simultaneous assessment of *p*-coumaric acid consumption and *p*-coumaroyl-CoA production.

After initial biosensor development and characterization, we demonstrated the applicability of CouR for dynamic regulation of the naringenin synthetic pathway in combination with another transcriptional repressor, FapR. We found *p*-coumaroyl-CoA accumulation to induce growth inhibition in the cells. Similar observations have been made in bacteria previously^50,51^ but have (to our knowledge) not been reported in yeast. The mechanism by which *p*-coumaroyl-CoA imposes growth hindrance is unknown. Nonetheless, we found that viability could be restored by adding the downstream *p*-coumaroyl-CoA consumption pathway by introducing CHS and CHI activity for naringenin biosynthesis. By the same token, the balancing of *p*-coumaroyl-CoA production through *4CL* regulation in response to malonyl-CoA and *p*-coumaroyl-CoA enhanced naringenin production, presumably by reducing the metabolic load caused by constant *4CL* expression and by mitigating *p*-coumaroyl-CoA toxicity. It should be noted that the regulated strains displayed larger variations in final titers than the nonregulated strain, which is expected due to an additional level of fluctuation caused by FapR and CouR transcriptional regulation and the observed binary behavior of CouR within each cell population. Additionally, the regulated strains exhibited superior naringenin/byproduct ratios, indicating that dynamic control of *p*-coumaroyl-CoA production may prompt less *p*-coumaroyl-CoA to be reduced to *p*-dihydrocoumaroyl-CoA. If accumulation was not controlled, more *p*-coumaroyl-CoA would be consumed by the competing reaction catalyzed by Tsc13 due to the limited activity of CHS.^16^ The supplementation of malonate to increase malonyl-CoA supply increased naringenin production and product/byproduct ratio in the nonregulated strain, suggesting malonyl-CoA as a bottleneck in this strain. The malonyl-CoA deficiency possibly leads to increased byproduct formation. Interestingly, malonate addition did not affect the FapR- and FapR-/CouR-regulated strains, indicating that endogenous malonyl-CoA supply via Acc1 was sufficient in these strains.

There are different strategies for the improvement of natural product synthesis in microbial cell factories, including coculturing and dynamic pathway control. As shown in this example, metabolite biosensors may not only be used for screening applications but can also be employed for the regulation of relevant pathways. This approach can be particularly beneficial for reducing the build-up of toxic intermediates. As *p*-coumaroyl-CoA is an essential precursor for a myriad of flavonoids and other phenylpropanoid compounds, we hope that this biosensor can become a useful tool for future studies.

## Materials and Methods

### Chemicals and Reagents

Oligonucleotide primers were synthesized by Eurofins Genomics Germany GmbH (Ebersberg, Germany) or Integrated DNA Technologies (Coralville, IA, USA). GeneJET Gel Extraction and Plasmid Miniprep kits were used for DNA purification (Thermo Fisher Scientific, Waltham, MA, USA). The Gibson assembly master mix was purchased from New England Biolabs (Ipswich, MA, USA). DNA fragments for plasmid construction were amplified by PCR using Phusion HF (New England Biolabs, Ipswich, MA, USA) or PrimeStar HS DNA polymerase (Takara Bio, Kusatsu, Shiga, Japan). DreamTaq DNA polymerase (Thermo Fisher Scientific, Waltham, MA, USA) was used for colony PCR. *CouR*, *RtMatB*, and *SpMae1* gene sequences were codon-optimized and synthesized by Doulix (Explora, Venice, Italy). *4CL* from *Arabidopsis thaliana* was amplified from pCfB854.^58^ The genes *CHS* from *Rhododendron simsii* and *CHI* from *Paeonia suffruticosa* were codon-optimized and synthesized by GenScript Biotech (Piscataway Township, NJ, USA). Analytical standards of naringenin (≥95%, TLC), malonic acid (≥98.5%, GC), phloretic acid (≥97.5%, HPLC), and *p*-coumaric acid (≥98%, HPLC) were obtained from Sigma-Aldrich/Merck KGaA (Darmstadt, Germany). Phloretin (≥99%, HPLC) was obtained from Extrasynthese (Lyon, France).

### Media and Culture Conditions

All chemicals for media preparation were purchased from Sigma-Aldrich/Merck, except for the yeast nitrogen base and complete supplement uracil dropout mixture, which were purchased from Formedium (Norfolk, United Kingdom). *E. coli* DH5α was routinely used for plasmid construction and propagation and grown in lysogeny broth (LB) made of 10 g/L peptone from casein, 5 g/L yeast extract, and 10 g/L NaCl. 100 mg/L ampicillin was added to LB medium for plasmid selection. Yeast peptone dextrose (YPD) medium containing 20 g/L yeast peptone from meat, 10 g/L yeast extract, and 20 g/L glucose was used for the preparation of competent yeast cells. In addition, 16 g/L or 20 g/L agar was added to make solid LB + ampicillin and YPD media, respectively. Delft medium^59^ (7.5 g/L (NH_4_)_2_SO_4_, 14.4 g/L KH_2_PO_4_, 0.5 g/L MgSO_4_·7H_2_O, 20 g/L glucose, 1 mL/L vitamin solution, and 2 mL/L trace metal solution), adjusted to pH 5.5 with 10 M KOH and supplemented with appropriate amino acids (76 mg/L l-histidine and/or 76 mg/L uracil), was used for cultivating yeast cells for flow cytometry and shake flask fermentation experiments. The vitamin solution consisted of 50 mg/L D-biotin, 200 mg/L *p*-aminobenzoic acid, 1 g/L nicotinic acid, 1 g/L d-pantothenic acid hemicalcium salt, 1 g/L pyridoxine-HCl, 1 g/L thiamine-HCl, and 25 g/L myo-inositol; the trace metal solution contained 4.5 g/L CaCl_2_·2H_2_O, 4.5 g/L ZnSO_4_·7H_2_O, 3 g/L FeSO_4_·7H_2_O, 1 g/L H_3_BO_3_, 1 g/L MnCl_2_·4H_2_O, 0.4 g/L Na_2_MoO_4_·2H_2_O, 0.3 g/L CoCl_2_·6H_2_O, 0.3 g/L CuSO_4_·5H_2_O, 0.1 g/L KI, and 19 g/L Na_2_EDTA·2H_2_O. *S. cerevisiae* and *E. coli* were cultured at 30 and 37 °C, respectively. Shake flask fermentation experiments for naringenin production under *p*-coumarate and malonate supplementation were carried out using Delft medium containing histidine and uracil. Precultures were grown overnight in 3 mL of media in 14 mL culture tubes and inoculated at a starting OD_600_ of 0.1 in 20 mL of fresh medium in 100 mL unbaffled shake flasks the next day. Cells were grown for 4 d at 220 rpm shaking. *p*-Coumarate (0.75 mg) in absolute ethanol (18.8 μL of 40 mg/L stock solution) and 3 mg of malonate in water (28.8 μL of 104 g/L stock solution) were added every 12 h. Cell culture (2 mL) was taken for HPLC measurements every 24 h.

### Plasmid and Strain Construction

All oligonucleotide primers, plasmids, and strains are listed in the Supporting Information (Tables S3–S6). Plasmid pX&Y19^49^ was used as a template for P_*CCW12*_BS_2_ sensor plasmids (pDL016-17 and pDL30-31), while p416TEF-GFP^38^ was used for constructing P_*TEF1*_BS_123_ sensor plasmids (pDL14-15 and pDL32-33). CouR DNA binding sites were inserted into the promoter sequence by whole-plasmid PCR, except for BS_2_ and BS_3_ in P_*TEF1*_, which were ordered as 120 bp oligos (DL086-87) and inserted by Gibson assembly (New England Biolabs, Ipswich, MA, USA). The RjCouR and RpCouR expression cassettes were also assembled using Gibson. All plasmids were verified by restriction digestion using appropriate FastDigest enzymes (ThermoFisher Scientific, Waltham, MA, USA) and Sanger sequencing (Eurofins Genomics Germany GmbH, Ebersberg, Germany). Negative and positive control plasmids included p416TEF,^60^ p416TEF-GFP,^38^ and p416CCW12-GFP (pDL103). All integrative plasmids were assembled using Gibson cloning into EasyClone-Markerfree vectors,^61^ which were linearized by digestion using suitable FastDigest restriction enzymes (ThermoFisher Scientific, Waltham, MA, USA). The naringenin pathway expression cassettes for constructing NAG10, NAG1-3, and NAG3-1 were made by fusion PCR of DNA fragments using PrimeStar DNA polymerase (primers listed in Supporting Information, Table S6). The same cassettes were used as templates for constructing integrative plasmids pMS01-04. For pMS07-09, the FapR sequence was amplified from pFDA09,^38^ while the modified *CCW12* promoter and the RpCouR expression cassette were amplified from pDL031. Native promoters and terminators were amplified from CEN.PK113-11C genomic DNA unless otherwise specified.

The *S. cerevisiae* strain CEN.PK113-11C (*MAT*a *ura3-52 his3*Δ *MAL2-8C SUC2*) was used as background strain for the evaluation of the biosensor’s maximum dynamic range. CEN.PK113-11C was also used as a basis for construction of strains yFlav06, yFlav13, and yMS04-06. The EasyClone-Markerfree kit was used for the integration of expression vectors into the genome.^61^ The *natMX* marker included in the original EasyClone-Markerfree gRNA plasmids was exchanged for a *URA3* marker to facilitate plasmid removal by growth on 5-fluoroorotic acid plates (6.9 g/L yeast nitrogen base without amino acids, 0.77 g/L complete supplement mix dropout -URA, 50 mg/L uracil, 1 g/L 5-fluoroorotic acid, 20 g/L glucose, and 20 g/L agar) after transformation. pDL006 was integrated into chromosomal locus XI-1 (gRNA plasmid pDL057) to obtain strain yFlav06. pDL038 was integrated into locus XII-5 (gRNA plasmid pDL060) to obtain strain yFlav13. yFlav06 was used as a background strain for the construction of yMS04-06. First, constructs pMS01-03 were integrated into loci X-2, XI-5, and XII-4 using triple gRNA plasmid pDL120. Then, pMS04 and pMS09 were sequentially integrated into loci X-4 (gRNA plasmid pDL056) and XII-5 to obtain yMS04. For yMS05 and yMS06, constructs pMS07 and pMS08 were integrated separately into locus XII-1 (gRNA plasmid pDL074) of yMS04. Strains NAG10, NAG1-3, and NAG3-1 used for evaluating the derepression behavior of the biosensor were based on the *p*-coumaric acid producer strain QL11.^15^ The gRNA plasmids and homology arms used have been published previously.^15^

Chemically competent DH5α *E. coli* cells were transformed with plasmids using heat shock.^62^ The lithium acetate method was used for all yeast transformations.^63^ For genomic integrations in strains derived from CEN.PK113-11C, the strain had to be transformed with the Cas9 expression plasmid (pCfB2312) first. The integrative plasmids were linearized using the restriction enzyme SmiI before genomic integration. SD-URA + G418 plates (6.9 g/L yeast nitrogen base without amino acids, 0.77 g/L complete supplement mix dropout -URA, 20 g/L glucose, 20 g/L agar, and 200 mg/L geneticin) were used for selection of CEN.PK113-11C transformants. QL11 transformants were selected on SD-URA plates (6.9 g/L yeast nitrogen base without amino acids, 0.77 g/L complete supplement mix dropout -URA, 20 g/L glucose, and 20 g/L agar). gRNA plasmids and pCfB2312 were removed prior to further strain evaluation. Successful integrations were verified by colony PCR. All biosensor plasmids carried a *URA3* marker, and transformants were selected on SD-URA plates.

### Growth Rate Measurement

To determine the growth rates of different strains, precultures were grown in 250 μL of Delft medium with appropriate amino acid supplementation in 96-well plates at 30 °C, 250 rpm shaking. Main cultures were grown in 250 μL of the same medium and under identical conditions, aiming for a starting OD_600_ of 0.1. A Growth Profiler 960 (Enzyscreen BV, Heemstede, the Netherlands) was used to compute OD_600_ values every 30 min.

### Fluorescence Measurement

Cells were cultured in Delft medium with 76 mg/L l-histidine. Precultures were grown in 2 mL of medium in 14 mL culture tubes at 30 °C overnight, shaking at 200 rpm. They were then diluted to an OD_600_ of 0.1 in 3 mL of medium in 14 mL cell culture tubes. Samples for fluorescence measurements were taken during exponential growth, by diluting to an OD_600_ of 0.02 in sterile water to aim for a cell count of <500 cells/μL. Green fluorescence was measured using a Guava easyCyte 6HT-2L flow cytometer (Luminex, s-Hertogenbosch, the Netherlands) with an excitation wavelength of 488 nm and a 525/30 BP filter. A total of 5000 events were acquired from each sample. Gain values were set to FSC: 4.36, SSC: 2.48, and GRN-B: 2.95. FlowJo Version 10 software (FlowJo LLC, Ashland, OR, USA) was used for analysis. The median intensity of the log-scale GFP fluorescence was used as a parameter for comparison between samples.

### Metabolite Extraction and Quantification

To measure metabolites of the naringenin production strains, the whole cell culture was used for sample preparation. Fermentation samples were freeze-dried, and metabolites were extracted with absolute ethanol by vortexing for 10 min and taking the supernatant. For samples used to characterize the derepression behavior of the biosensor, metabolites were instead extracted directly by adding an equal volume of absolute ethanol to the cell culture, vortexing for 10 min, and saving the supernatant. Samples were analyzed using a Dionex UltiMate 3000 HPLC (ThermoFisher Scientific, Waltham, MA, USA) configured with a UVD 340U UV/VIS diode array detector (ThermoFisher Scientific, Waltham, MA, USA) and a Discovery HS F5 column (15 cm × 4.6 mm, 5 μm particle size) (Sigma-Aldrich, St. Louis, MO, USA). A gradient elution program was applied at a flow rate of 1.2 mL/min, using acetonitrile (B) and 10 mM ammonium formate, pH 3 (A). The eluent gradient started with 15% B (0–1.5 min), followed by an increase to 20% B (1.5–3 min), 25% B (3–24 min), 45% B (24–25 min), and 50% B (25–27 min) and a final decrease back to 15% B (27–28 min). The sample injection volume was set to 10 μL, and the column department was kept at 30 °C. All compounds were detected at a wavelength of 280 nm at retention times of approximately 4.9 min (*p*-coumaric acid), 5.5 min (phloretic acid), 14.2 min (naringenin), and 14.7 min (phloretin)..

## Supporting Information Available

Growth curves of wild-type and *4CL*-expressing strain upon *p*-coumaric acid addition; growth rates of wild-type strain transformed with biosensor and control plasmids; plasmid loss in wild-type strain carrying RjCouR or RpCouR expression cassette; overlaid histograms of individual clones of QL11, NAG1-3, NAG10, and NAG3-1 carrying the RjCouR or RpCouR biosensor plasmid; biosensor response to other compounds in the naringenin biosynthetic pathway; metabolite concentrations in naringenin strains QL11, NAG1-3, NAG10, and NAG3-1; metabolite concentrations in yMS04, yMS05, and yMS06; naringenin concentration profiles of yMS04, yMS05, and yMS06 over 4 d of cultivation; naringenin/byproduct ratios in strains yMS04, yMS05, and yMS06; modified promoter and heterologous gene sequences used in this study; and primers, plasmids, and strains used in this study (PDF)
