# Supplementary material for: A p-Coumaroyl-CoA Biosensor for Dynamic Regulation of Naringenin Biosynthesis in Saccharomyces cerevisiae
Source: ACS Synth Biol. 2022 Sep 22;11(10):3228–38. doi: 10.1021/acssynbio.2c00111 (PMC9594313; doi:10.1021/acssynbio.2c00111)
Supplement: Supplementary file 1 — sb2c00111_si_001.pdf [file sb2c00111_si_001.pdf]

Supporting Information

A *p*-Coumaroyl-CoA biosensor for Dynamic Regulation of Naringenin Biosynthesis in *Saccharomyces cerevisiae*

Dany Liu,[1] Maria Sole Sica,[1] Jiwei Mao,[1] Lucy Fang-I Chao,[1] Verena Siewers[1*]

1. Department of Biology and Biological Engineering, Chalmers University of Technology, SE-412 96 Gothenburg, Sweden.

*Corresponding author: siewers@chalmers.se

Contents

Figure S1. Growth curves of wild-type CEN.PK113-11C and *4CL* expressing strain yFlav13 under *p*-coumaric acid addition. S2

Figure S2. Growth rates of the CEN.PK113-11C wild-type strain transformed with biosensor or control plasmids. S3

Figure S3. Plasmid loss in CEN.PK113-11C carrying an empty plasmid (p416TEF) or an RjCouR/RpCouR expressing plasmid (pDL030/pDL031). S4

Figure S4. Overlaid histograms of individual clones of QL11 (0:0:0) , NAG1-3 (1:3:3) , NAG10 (1:1:1) and NAG3-1 (3:1:1) carrying the CouR biosensor in two independent experiments. S5

Figure S5. Biosensor response to other compounds involved in the naringenin biosynthetic pathway. S6

Figure S6. Naringenin, *p*-coumaric acid, phloretic acid and phloretin concentrations in strains QL11, NAG1-3, NAG10 and NAG3-1 with different copy numbers of *4CL*, *CHS* and *CHI*. S7

Figure S7. Naringenin, *p*-coumaric acid, phloretic acid and phloretin concentrations in the non-regulated (yMS04) and FapR- and FapR-/CouR-regulated strains (yMS05, yMS06) after 3 d. S8

Figure S8. Naringenin concentration profile over 4 d of cultivation. S9

Figure S9. Naringenin:byproduct ratios in the three production strains yMS04, yMS05 and yMS06 (non-regulated, FapR-regulated and FapR-/CouR-regulated) after 3 d of cultivation. S10

Table S1. Sequences of modified promoters used in this study. S11

Table S2. Codon-optimized heterologous gene sequences used in this study. S12

Table S3: Oligonucleotide primers used in this study. S14

Table S4: Oligonucleotide primers used for construction of naringenin strains QL11, NAG1-3, NAG10 and NAG3-1. ....S17

Table S5: Plasmids used in this study. S20

Table S6: Strains used in this study. S22

References S23

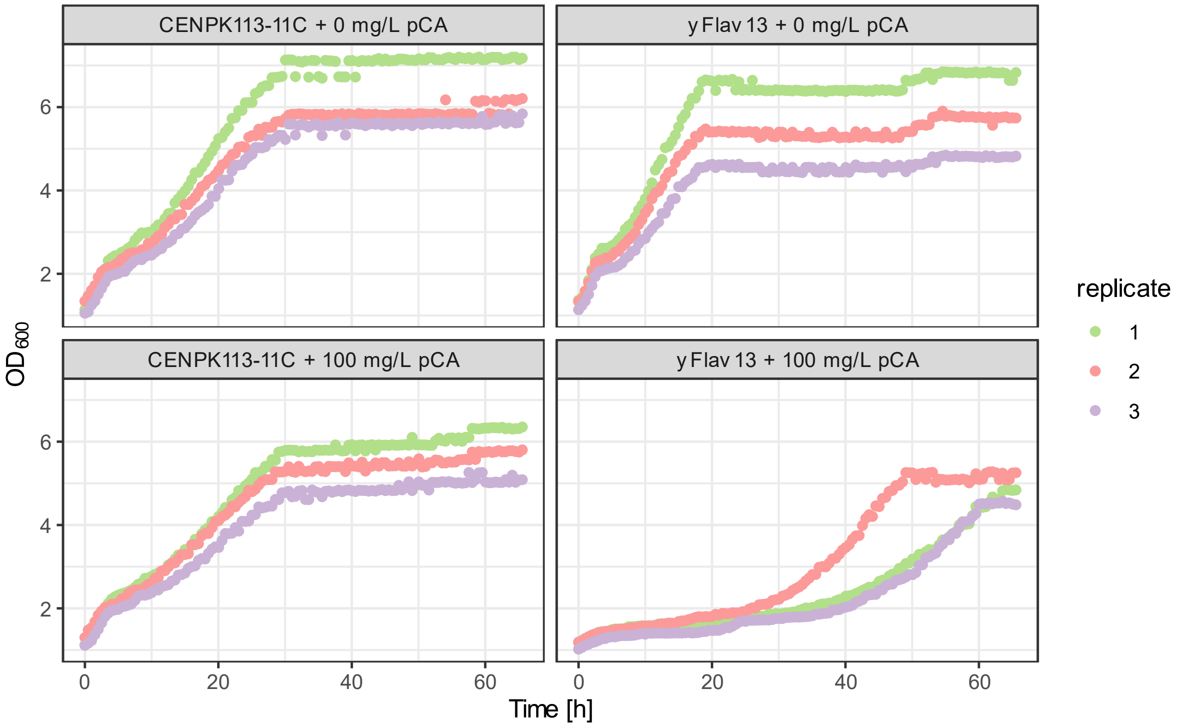

Figure S1. Growth curves of wild-type CEN.PK113-11C and *4CL* expressing strain yFlav13 under *p*-coumaric acid addition. Cells were grown in 250 µL Delft medium with histidine and uracil supplementation in 96-well plates, with 0 mg/L or 100 mg/L *p*-coumaric acid at 30°C, 250 rpm shaking. OD_{600} values were obtained using a growth profiler.

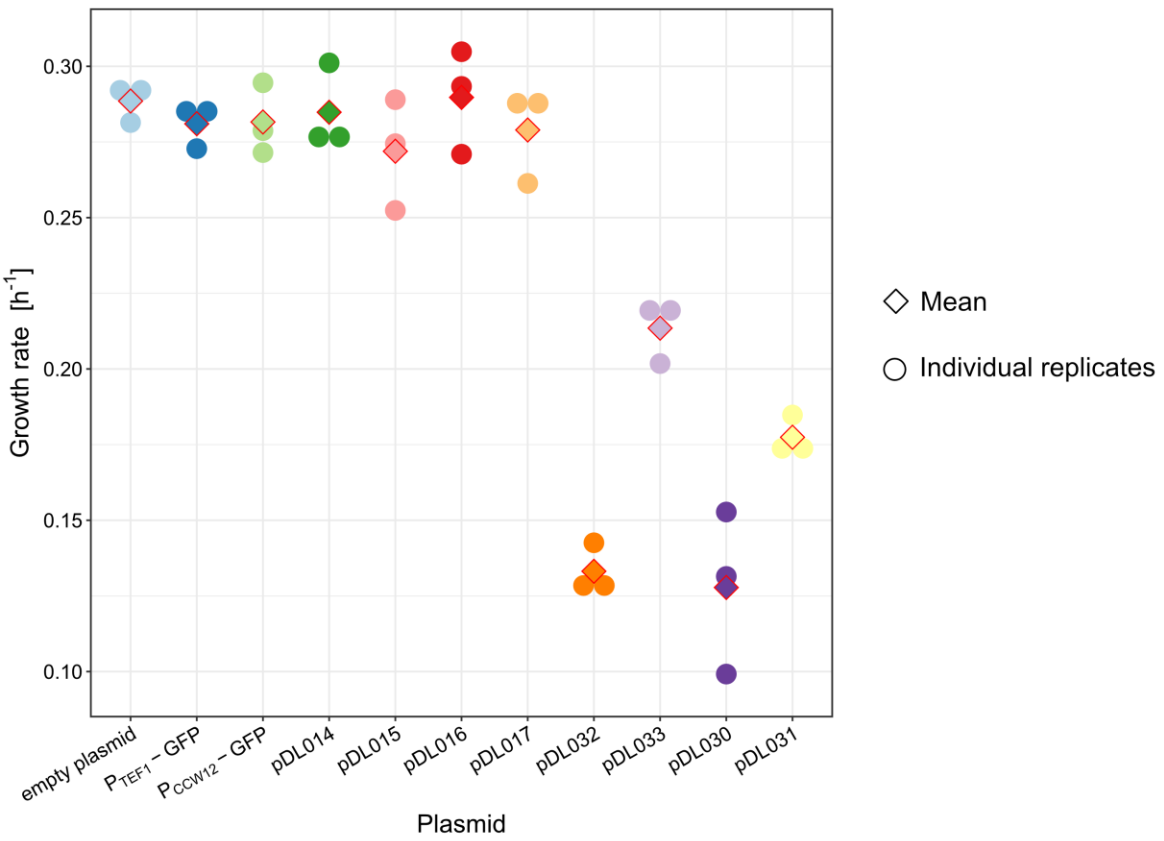

Figure S2. Growth rates of the CEN.PK113-11C wild-type strain transformed with biosensor or control plasmids. pDL014-pDL017 contain only the GFP expression cassettes with the modified P_{TEF1} or P_{CCW12} promoter. pDL30 and pDL032 contain the GFP and the RjCouR expression cassettes, while pDL031 and pDL033 contain the GFP and RpCouR expression cassettes. Detailed plasmid descriptions are given in Table S5. Cells were grown in 250 µL Delft medium with histidine supplementation in 96-well plates at 30°C, 250 rpm shaking. OD_{600} values were obtained using a growth profiler. The experiment was carried out in biological triplicates.

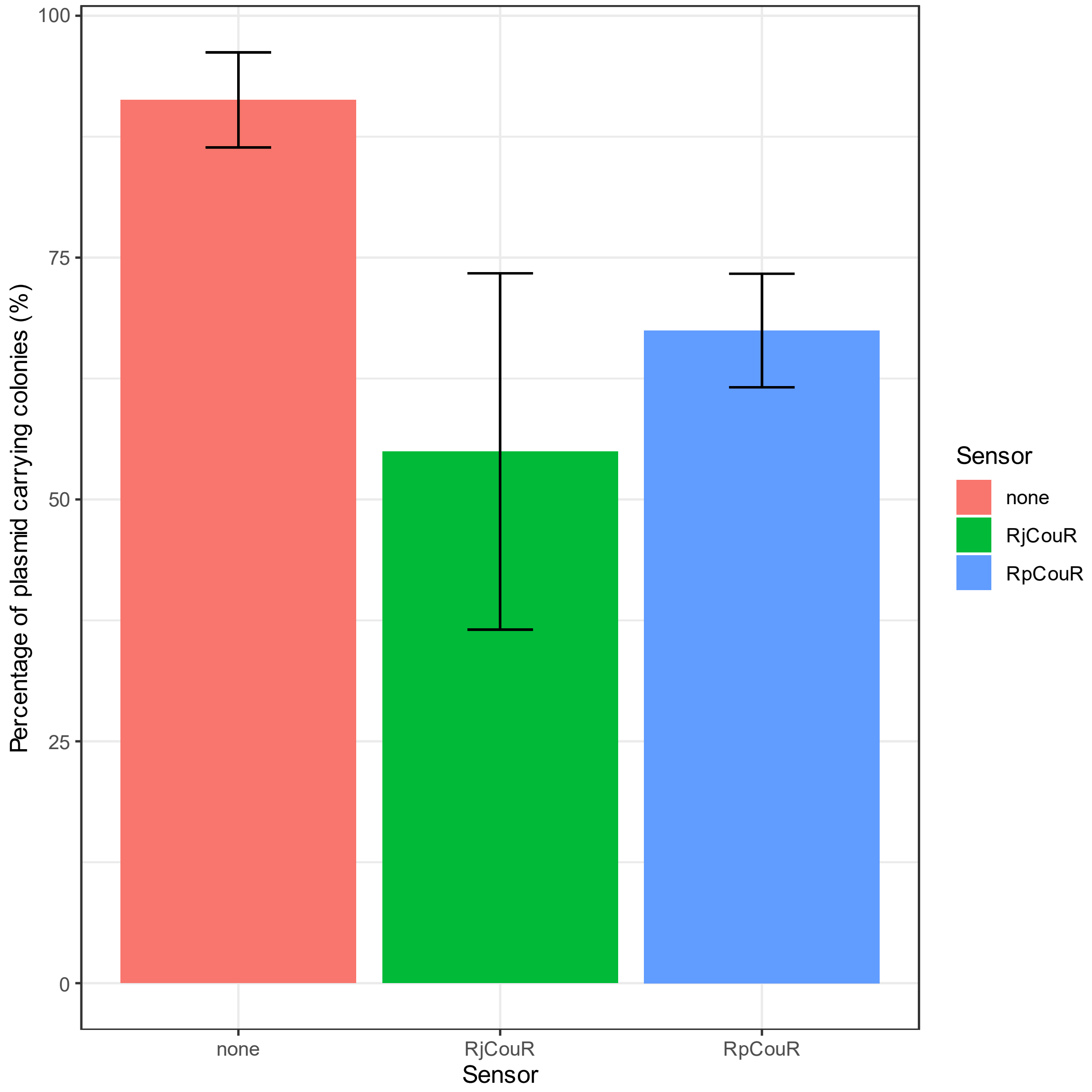

Figure S3. Plasmid loss in CEN.PK113-11C carrying an empty plasmid (p416TEF) or an RjCouR/RpCouR expressing plasmid (pDL030/pDL031). Cells were cultured in Delft medium with histidine supplementation overnight. Cells were then diluted to ca. 1.5 10^3 cells/mL and 100 µL was plated on non-selective synthetic defined (SD) medium and selective dropout medium (SD-URA). Colonies were counted after 3 days of growth at 30°C. Percentages of plasmid-carrying colonies were calculated by dividing the number of colonies on SD-URA plates by the number of colonies on SD plates.

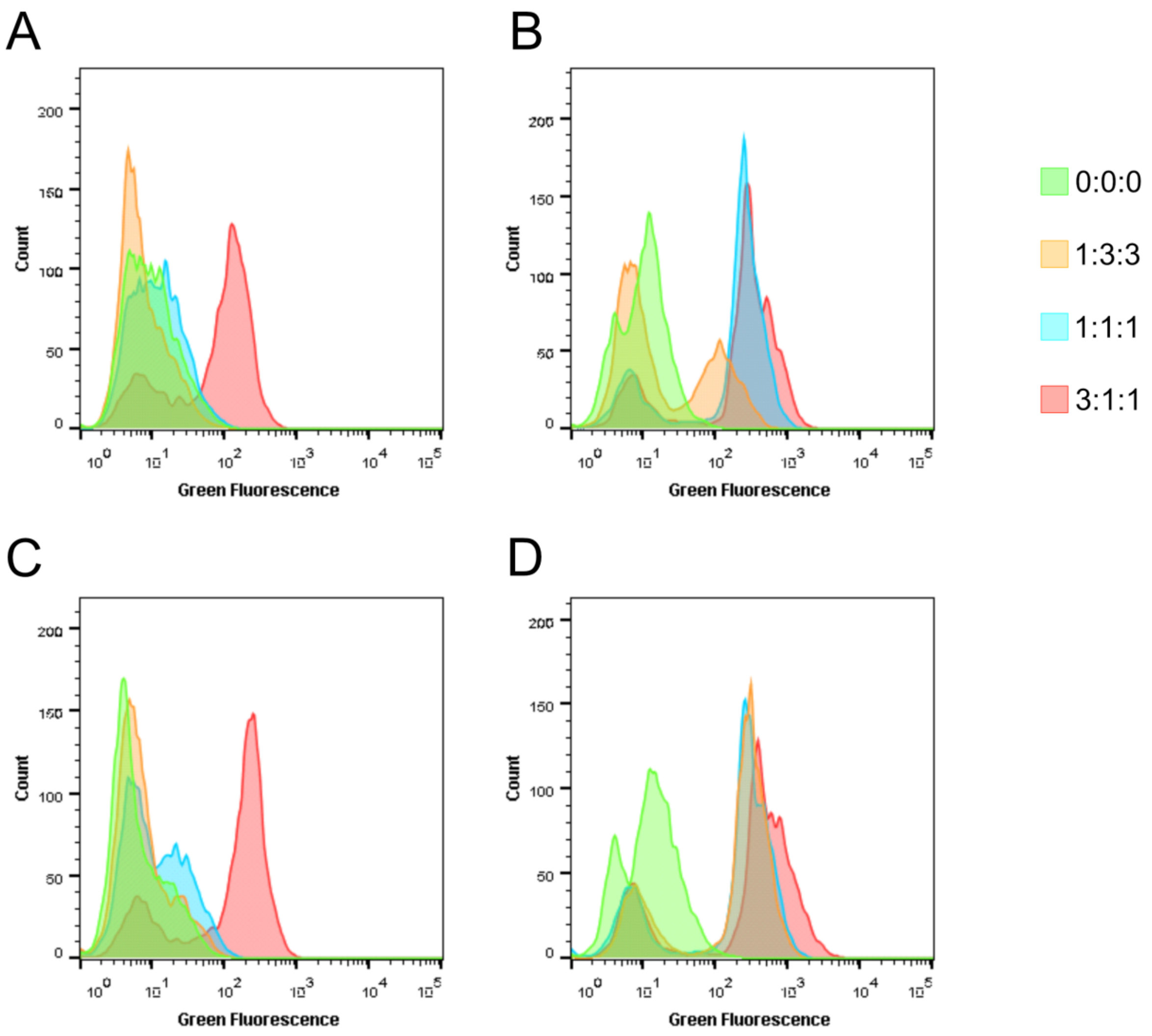

Figure S4. Overlaid histograms of individual clones of QL11 (0:0:0) **, NAG1-3** (1:3:3) **, NAG10** (1:1:1) **and NAG3-1** (3:1:1) **carrying the CouR biosensor** in two independent experiments**. (A)** RjCouR biosensor, experiment 1. **(B)** RpCouR biosensor, experiment 1. **(C)** RjCouR biosensor, experiment 2. **(D)** RpCouR biosensor, experiment 2. Strains were cultivated in 3 mL Delft medium and samples were taken after 18 h of growth at 30°C, 200 rpm shaking. Fluorescence intensities of 5000 cells were measured for each sample using flow cytometry.

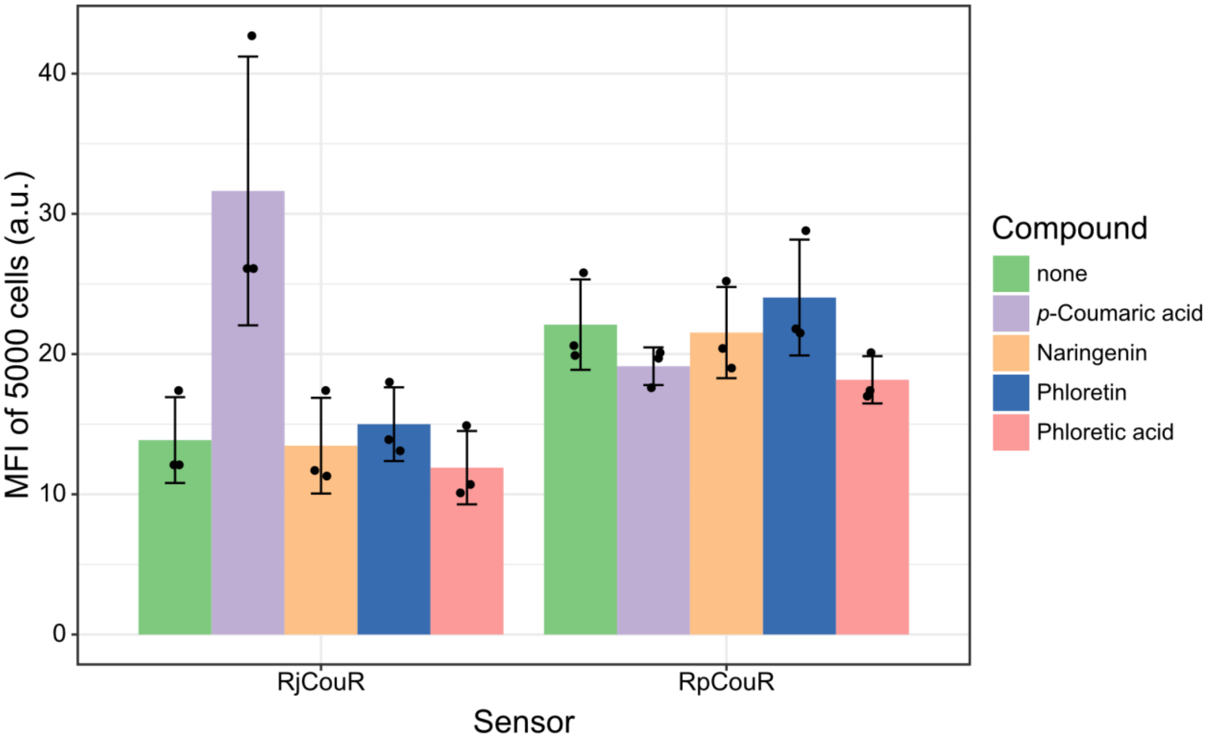

Figure S5. Biosensor response to other compounds involved in the naringenin biosynthetic pathway. CEN.PK113-11C carrying biosensor plasmids pDL030 (RjCouR) or pDL031 (RpCouR) were cultured in 2 mL Delft medium with histidine supplementation. Highest soluble concentrations of *p*-coumaric acid (6.1 mM), naringenin (275 µM), phloretin (274 µM) and phloretic acid (12 mM) were tested. The median fluorescence of 5000 cells was determined by flow cytometry 18 h after induction. The experiment was carried out in biological triplicates.

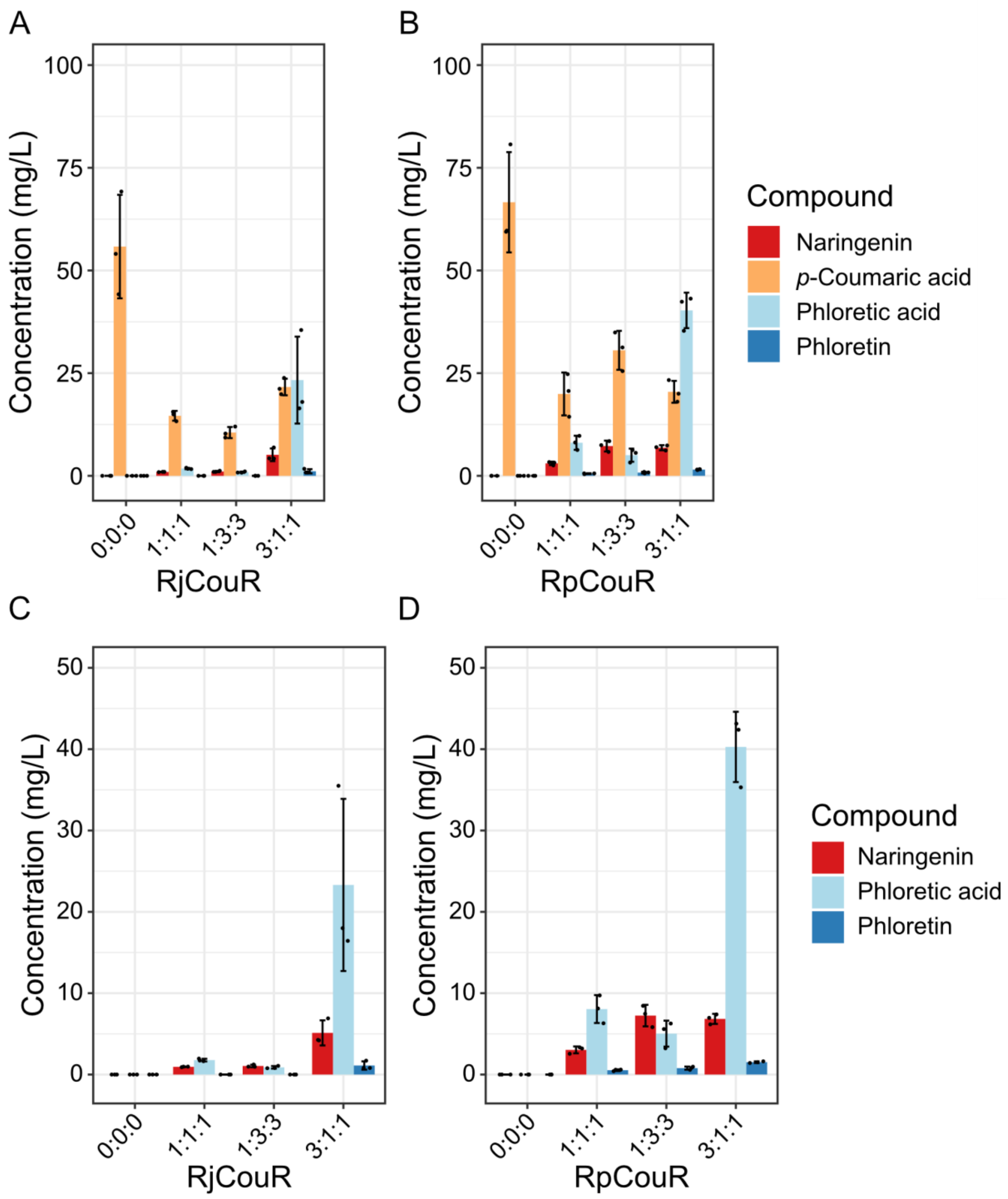

Figure S6. Naringenin, *p*-coumaric acid, phloretic acid and phloretin concentrations in strains QL11, NAG1-3, NAG10 and NAG3-1 with different copy numbers of *4CL*, *CHS* and *CHI*. (A) Metabolite concentrations in cells equipped with the RjCouR biosensor. **(B)** Metabolite concentrations in cells expressing the RpCouR biosensor. **(C, D)** Zoom in on naringenin, phloretic acid and phloretin concentrations. Cells were grown in 3 mL Delft medium for 18 h at 30°C, 200 rpm shaking and sampled for flow cytometry and HPLC measurements. Error bars represent standard deviation.

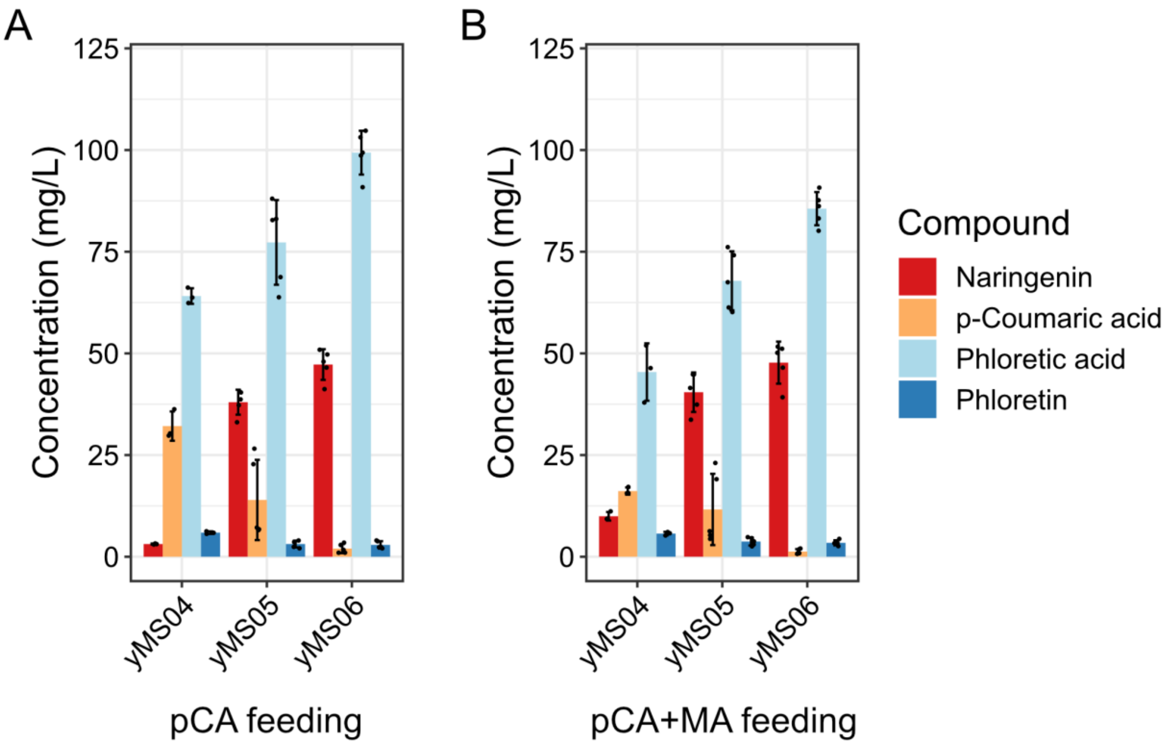

Figure S7. Naringenin, *p*-coumaric acid, phloretic acid and phloretin concentrations in the non-regulated (yMS04) and FapR- and FapR-/CouR-regulated strains (yMS05, yMS06) after 3 d. Cells were grown in 20 mL Delft medium with histidine and uracil supplementation for 4 d at 30°C, 220 rpm shaking. **(A)** 0.75 mg *p*-coumaric acid was added every 12 h. **(B)** 0.75 mg *p*-coumaric acid and 3 mg malonic acid were added every 12 h. Error bars represent standard deviation.

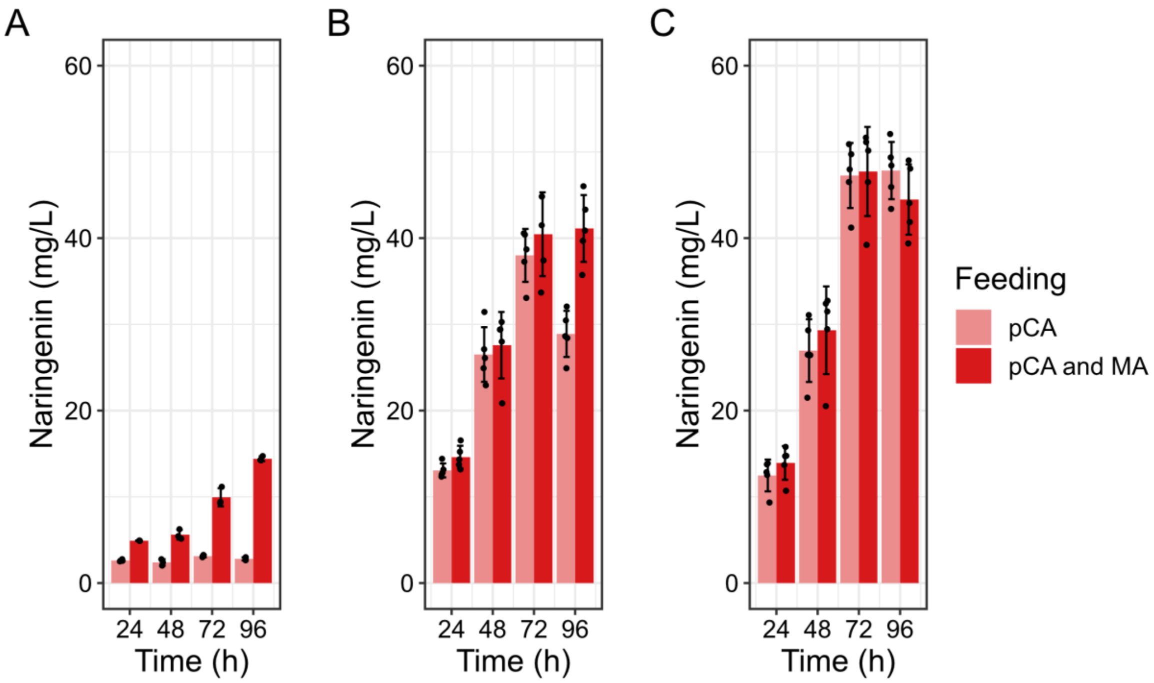

Figure S8. Naringenin concentration profile over 4 d of cultivation. (A) Non-regulated naringenin production strain yMS04 with four copies of *CHS* and *CHI* and one copy of *4CL*. **(B)** FapR-regulated naringenin production strain yMS05. **(C)** FapR- and CouR-regulated strain yMS06. Cells were cultured in 20 mL Delft medium with histidine and uracil supplementation at 30°C, 220 rpm shaking. 0.75 mg *p*-coumarate in absolute ethanol (18.8 µL of 40 mg/L stock solution) and 3 mg malonate in water (28.8 µL of 104 g/L stock solution) was added every 12 h. Error bars represent standard deviation.

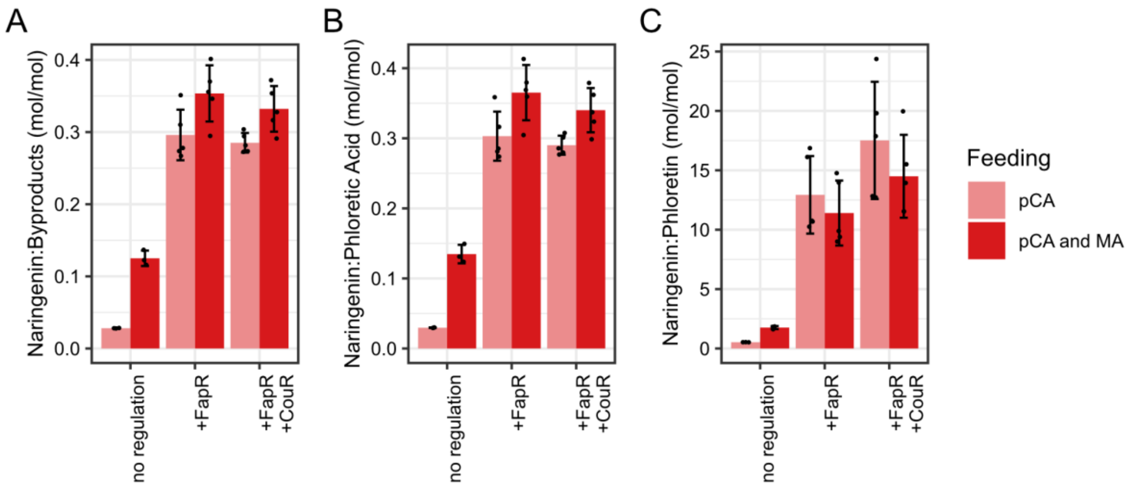

Figure S9. Naringenin:byproduct ratios in the three production strains yMS04, yMS05 and yMS06 (non-regulated, FapR-regulated and FapR-/CouR-regulated) after 3 d of cultivation. (A) Naringenin:(phloretin+phloretic acid) molar ratio. **(B)** Naringenin:phloretic acid molar ratio. **(C)** Naringenin:phloretin molar ratio. Cells were cultured in 20 mL Delft medium with histidine and uracil supplementation at 30°C, 220 rpm shaking. 0.75 mg *p*-coumarate in absolute ethanol (18.8 µL of 40 mg/L stock solution) and 3 mg malonate in water (28.8 µL of 104 g/L stock solution) was added every 12 h. Error bars represent standard deviation. Error bars represent standard deviation.

Table S1. Sequences of modified promoters used in this study.

**P_{CCW12}BS(RjCouR)**	CGACACGCAAAAGAAAACCTTCGAGGTTGCGCACTTCGCCCACCCATGAACCACACGGTTA<br>GTCCAAAAGGGGCAGTTCAGATTCCAGATGCGGGAATTAGCTTGCTGCCACCCTCACCTCA<br>CTAACGCTGCGGTGTGCGGATACTTCATGCTATTTATAGACGCGCGTGTCGGAATCAGCAC<br>GCGCAAGAACCAAATGGGAAAATCGGAATGGGTCCAGAACTGCTTTGAGTGCTGGCTATTG<br>GCGTCTGATTTCCGTTTTGGGAATCCTTTGCCGCGCGCCCCTCTCAAAACTCCGCACAAGTC<br>CCAGAAAGCGGGAAAGAAATAAAACGCCACCAAAAAAAAAAATAAAAGCCAATCCTCGAAGC<br>GTGGGTGGTAGGCCCTGGATTATCCCGTACAAGTATTTCTCAGGAGTAAAAAAACCGTTTGT<br>TTTGGAATTCCCCATTTCGCGGCCACCTACGCCGCTATCTTTGCAACAACTATCTGCGATAA<br>CTCAGCAAATTTTGCATATTCGTGTTGCAGTATTGCGATAATGGGAGTCTTACTTCCAACATA<br>ACGGCAGAAAGAAATGTGAGAAAATTTTGCATCCTTTGCCTCCGTTCAAGTATATAAAGTCG<br>GCACATTGATGATTGAGAATGTCAATGATTAAGGTGCTTGATAATCTTTCTTTCCATCCTACA<br>TTGTTCTAATTATTCTTATTCTCCTTTATTCTTTCCTAACATACCAAGAAATTAATCTTCTGTCA<br>TTCGCTTAAACACTATATCAATA
**P_{CCW12}BS(RpCouR)**	CGACACGCAAAAGAAAACCTTCGAGGTTGCGCACTTCGCCCACCCATGAACCACACGGTTA<br>GTCCAAAAGGGGCAGTTCAGATTCCAGATGCGGGAATTAGCTTGCTGCCACCCTCACCTCA<br>CTAACGCTGCGGTGTGCGGATACTTCATGCTATTTATAGACGCGCGTGTCGGAATCAGCAC<br>GCGCAAGAACCAAATGGGAAAATCGGAATGGGTCCAGAACTGCTTTGAGTGCTGGCTATTG<br>GCGTCTGATTTCCGTTTTGGGAATCCTTTGCCGCGCGCCCCTCTCAAAACTCCGCACAAGTC<br>CCAGAAAGCGGGAAAGAAATAAAACGCCACCAAAAAAAAAAATAAAAGCCAATCCTCGAAGC<br>GTGGGTGGTAGGCCCTGGATTATCCCGTACAAGTATTTCTCAGGAGTAAAAAAACCGTTTGT<br>TTTGGAATTCCCCATTTCGCGGCCACCTACGCCGCTATCTTTGCAACAACTATCTGCGATAA<br>CTCAGCAAATTTTGCATATTCGTGTTGCAGTATTGCGATAATGGGAGTCTTACTTCCAACATA<br>ACGGCAGAAAGAAATGTGAGAAAATTTTGCATCCTTTGCCTCCGTTCAAGTATATAAAGTCG<br>GCATTGTTATACTCTATAACTATTCTGCACAGTGCTTGATAATCTTTCTTTCCATCCTACATTG<br>TTCTAATTATTCTTATTCTCCTTTATTCTTTCCTAACATACCAAGAAATTAATCTTCTGTCATTC<br>GCTTAAACACTATATCAATA
**P_{TEF1}_BS123(RjCouR)**	CAAAATGTTTCTACTCCTTTTTTACTCTTCCAGATTTTCTCGGACTCCGCGCATCGCCGTACC<br>ACTTCAAAACACCCAAGCACAGCATACTAAATTTCCCCTCTTTCTTCCTCTAGGGTGTCGTTA<br>ATTACCCGTACTAAAGGTTTGGAAAAGAAAAAAGAGACCGCCTCGTTTCTTTTTCTTCGTCGA<br>AAAAGGCAATAAAAATTTTTATCACGTTTCTTTTTCTTGAAAATTTTTTTTTTTGATTTTTTTCTC<br>TTTCGATGACCTCCCATTGATATTTAAGTTAATAAACGGTCTTCAATTTCTCAAGTTTCAGTTT<br>CATTTTTCTTGTTCCATTGATGATTGAGAATGTCAATGATTAAGGTATTACAACTTTTTTTACTT<br>CTTGCTCATTAGACATTGATGATTGAGAATGTCAATGATTAAGGAAGAAAGCACATTGATGAT<br>TGAGAATGTCAATGATTAAGGTAGCAATCTAATCTAAGTTTTAATTACAAA
**P_{TEF1}_BS123(RpCouR)**	CAAAATGTTTCTACTCCTTTTTTACTCTTCCAGATTTTCTCGGACTCCGCGCATCGCCGTACC<br>ACTTCAAAACACCCAAGCACAGCATACTAAATTTCCCCTCTTTCTTCCTCTAGGGTGTCGTTA<br>ATTACCCGTACTAAAGGTTTGGAAAAGAAAAAAGAGACCGCCTCGTTTCTTTTTCTTCGTCGA<br>AAAAGGCAATAAAAATTTTTATCACGTTTCTTTTTCTTGAAAATTTTTTTTTTTGATTTTTTTCTC<br>TTTCGATGACCTCCCATTGATATTTAAGTTAATAAACGGTCTTCAATTTCTCAAGTTTCAGTTT<br>CATTTTTCTTGTTCTTGTTATACTCTATAACTATTCTGCACAGTATTACAACTTTTTTTACTTCT<br>TGCTCATTAGATTGTTATACTCTATAACTATTCTGCACAGAAGAAAGCATTGTTATACTCTATA<br>ACTATTCTGCACAGTAGCAATCTAATCTAAGTTTTAATTACAAA

TATA Box
CouR DNA binding sequence
Transcription start site

Table S2. Codon-optimized heterologous gene sequences used in this study.

**Codon-optimized *RjCouR* sequence**	ATGGCTGAATCTCAAGCCTTGTCTGATGATATTGGTTTCTTGTTGTCAAGAGTTGGTGGTATG<br>GTTTTGGGTGCTGTTAACAAGGCTTTGGTTCCAACTGGTTTGAGAGTTAGATCTTACTCCGTT<br>TTGGTTTTGGCTTGTGAACAAGCTGAAGGTGTTAATCAAAGAGGTGTTGCTGCTACTATGGG<br>TTTAGATCCATCTCAAATAGTTGGTTTGGTCGACGAATTGGAAGAAAGAGGTTTGGTTGTTA<br>GAACTTTGGACCCATCTGATAGACGTAACAAATTGATTGCTGCAACCGAAGAAGGTAGAAGA<br>TTGAGAGATGATGCTAAGGCTAGAGTTGATGCTGCTCATGGTAGATACTTTGAAGGTATTCC<br>AGATACCGTTGTCAACCAGATGAGAGATACCTTGCAATCTATTGCTTTCCCAACCTTCGTTGA<br>G
**Codon-optimized *RpCouR* sequence**	ATGACCTCCTCTAACAGAATTACTTCTCCAGCTATGACTGCTTCTAAAACTGCTGCTGTTGCT<br>AAACCTACTAGAGCTGGTAGAAAAGCTCCAGCTGTTGAAACTGCTCCAGAAGCTTCAGAATT<br>GAAAATGGGTGAATTGTCTGAGTTGTTGGGTTACGCTTTGAAAAGAGCACAATTGAGGGTTT<br>TCGAAGATTTCTTGCATTGTGTTGCTCCAGTTCAATTGACTCCAGCTCAATTTTCTGTCTTGT<br>TGTTGTTGGATGCTAACCCAGGTAGAAATCAAACTGAAATTGCTACCACCTTGGGTATTTTGA<br>GGCCAAATTTTGTTGCTATGTTGGACGCTTTGGAAGGTAGAGGTTTGTGTGTTAGAACTAGA<br>TCTCCATCCGATAGAAGATCCCATATTTTGATGTTGACCGATAAGGGTAGAGCTACTTTGGC<br>TAGAGCTAAAAAGTTGGTTGCTACCAGACATGAAGATAGATTGACCGAATTATTGGGTAGAG<br>ATAACAGAGATGCCCTGTTGTCTATGTTAGCTACAATTGCCAGAGAGTTC
**Codon-optimized *At4CL1* sequence**	ATGGCTCCACAAGAACAAGCTGTTTCCCAAGTTATGGAAAAGCAATCTAACAACAACAACTC<br>CGACGTCATCTTCAGATCTAAATTGCCAGATATCTACATCCCAAACCACTTGTCATTGCACGA<br>TTACATCTTCCAAAACATCTCTGAATTCGCTACCAAGCCATGTTTGATTAACGGTCCAACTGG<br>TCATGTTTACACCTACTCTGATGTTCACGTTATCTCCAGACAAATTGCTGCTAACTTTCACAA<br>GTTGGGTGTCAATCAAAACGATGTCGTCATGTTGTTATTGCCAAACTGTCCAGAATTCGTCTT<br>GTCTTTTTTGGCTGCTTCTTTTAGAGGTGCTACTGCTACAGCTGCTAATCCATTTTTTACTCC<br>AGCTGAAATTGCTAAGCAAGCTAAGGCTTCTAACACCAAGTTGATTATTACCGAAGCTAGAT<br>ACGTTGACAAGATCAAGCCATTGCAAAATGATGATGGTGTTGTTATCGTCTGCATCGATGAT<br>AATGAATCCGTTCCAATTCCAGAAGGTTGTTTGAGATTCACTGAATTGACTCAATCTACTACC<br>GAAGCCTCCGAAGTTATTGATTCCGTTGAAATTTCTCCAGATGATGTTGTTGCTTTGCCATAC<br>TCTTCAGGTACTACTGGTTTGCCAAAAGGTGTTATGTTGACTCATAAGGGTTTGGTTACATCC<br>GTTGCTCAACAAGTTGATGGTGAAAATCCAAACTTGTACTTCCACTCCGATGATGTCATTTTG<br>TGTGTTTTGCCAATGTTCCATATCTACGCCTTGAACTCTATTATGTTGTGCGGTTTGAGAGTT<br>GGTGCTGCTATTTTGATTATGCCAAAGTTCGAAATCAATTTGTTGTTGGAATTGATCCAAAGA<br>TGCAAGGTTACTGTTGCTCCAATGGTTCCACCAATAGTTTTGGCTATTGCTAAGTCCTCTGAA<br>ACCGAAAAGTACGACTTGTCCTCTATCAGAGTTGTTAAGTCAGGTGCTGCTCCATTGGGTAA<br>AGAATTAGAAGATGCTGTTAACGCCAAGTTCCCAAATGCTAAATTGGGTCAAGGTTACGGTA<br>TGACTGAAGCTGGTCCAGTTTTAGCTATGTCTTTGGGTTTTGCTAAAGAACCATTCCCAGTAA<br>AATCTGGTGCTTGTGGTACAGTTGTTAGAAACGCTGAAATGAAGATCGTTGATCCAGATACT<br>GGTGACTCCTTGTCTAGAAATCAACCAGGTGAAATCTGCATCAGAGGTCATCAAATTATGAA<br>GGGTTACTTGAACAATCCAGCTGCTACTGCAGAAACCATTGATAAGGATGGTTGGTTGCATA<br>CAGGTGATATTGGTTTGATTGATGACGACGACGAATTATTCATCGTTGATAGATTGAAAGAAT<br>TGATCAAGTACAAGGGTTTCCAAGTTGCTCCAGCAGAATTGGAAGCTTTGTTGATTGGTCAT<br>CCAGATATTACCGATGTTGCTGTTGTTGCAATGAAGGAAGAAGCTGCTGGTGAAGTTCCAGT<br>TGCTTTTGTTGTCAAATCCAAGGACTCTGAATTGTCCGAAGATGATGTAAAGCAATTCGTCAG<br>TAAGCAAGTCGTTTTCTACAAGAGAATCAACAAGGTTTTCTTCACCGAATCCATTCCAAAAGC<br>TCCATCTGGTAAGATCTTGAGAAAAGATTTGAGAGCCAAGTTGGCTAACGGTTTGTGA
**Codon-optimized *RsCHS* sequence**	ATGGTTACTGTTGAAGATGTTAGAAGAGCACAAAGAGCTGAAGGTCCAGCAACAGTTATGGC<br>TATTGGTACTGCAACACCATCTAATTGTGTTGATCAATCAACTTACCCAGATTTCTACTTTAGA<br>ATTACAAATTCTGAACATAAGGCTGAATTAAAGGAAAAGTTCCAAAGAATGTGTGATAAATCA<br>ATGATTAAGAAAAGATATATGTACTTAACTGAAGAAATTTTGAAGGAAAACCCATCTGTTTGT<br>GAATATATGGCTCCATCATTAGATGCAAGACAAGATATGGTTGTTGTTGAAGTTCCAAAATTG<br>GGTAAAGAAGCTGCAACTAAAGCTATTAAAGAATGGGGTCAACCAAAGTCTAAGATCACACA<br>TTTGGTTTTCTGTACTACATCAGGTGTTGATATGCCAGGTGCAGATTACCAATTGACTAAATT<br>GTTGGGTTTAAGACCATCTGTTAAGAGATTGATGATGTACCAACAAGGTTGTTTTGCTGGTG<br>GTACAGTTTTGAGATTGGCTAAAGATTTGGCAGAAAACAATAAGGGTGCTAGAGTTTTGGTT<br>GTTTGTTCAGAAATCACTGCTGTTACTTTTAGAGGTCCATCTGATACACATTTGGATTCATTG<br>GTTGGTCAAGCATTGTTTGGTGACGGTGCTGCTGCTATTATTGTTGGTGCAGATCCAGTTCC<br>AGAAGTTGAAAAGCCATTGTTCGAATTGGTTTCTGCTGCTCAAACTATTTTACCAGATTCAGA<br>TGGTGCTATTGATGGTCATTTGAGAGAAGTTGGTTTGACATTCCATTTGTTGAAGGATGTTCC<br>AGGTTTGATCTCTAAAAATATCGAAAAAGCATTGACTGAAGCATTTCAACCATTGGGTATTTC<br>TGATTGGAACTCTATTTTCTGGATTGCTCATCCAGGTGGTCCAGCAATTTTGGATCAAGTTGA<br>ATTGAAATTATCTTTGAAGCCAGAAAAATTGAGAGCTACAAGACATGTTTTGTCAGAATACGG<br>TAACATGTCTTCAGCATGTGTTTTGTTTATTTTGGATGAAATGAGAAGAAAATCTGCTGAAGA<br>AGGTTTAAAAACTACAGGTGAAGGTTTAGAATGGGGTGTTTTGTTTGGTTTTGGTCCAGGTTT<br>GACTGTTGAAACAGTTGTTTTGCATTCATTGTGTACTTAA

**Codon-optimized *PsCHI* sequence**	ATGGCAAAACCACCATCTGTTTCAGGTGTTAACATCGAATCTTATGCTTTTCCACCAACTGTT<br>AAACCACCAGGTTCTACTAAAACATTGTTTTTAGGTGGTGCAGGTGTTAGAGGTTTGGAAGT<br>TCCAACTGGTCAATTCGTTAAGTTTACTGCTATCGGTGTTTACTTGGAAGATAACGCAATCAC<br>TTCATTAGCTGTTAAGTGGAAAGGTAAAACTGCTGAAGAATTAACAGAATCTGATGATTTCTT<br>TAGAGATATCGTTACAGGTCCATTCGAAAAGTTTACTCAAGTTACAATGATCTTGCCATTGAC<br>TGGTCAACAATACTCAGAAAAGGTTACAGAAAACTGTGTTGCTTACTGGAAAGCAGTTGGTG<br>CTTACACTGATGCTGAAGCATCTGCTATTGAAAAGTTTATTGAAGTTTTTAAAGATGAAAAGT<br>TTCCACCAGGTTCTTCAATTTTGTTTACTCAAACACCAGAAGGTTCTTTAACAATCGGTTTTTC<br>AAAGGATGGTGTTTTGCCAGAAGTTGGTAATGCAGTTGTTGAAAATAAGCAATTGTCTGAAG<br>CTGTTTTAGAATCAATCATCGGTAAACATGGTGTTTCTCCAGAAGCTAAACAATCATTAGCTG<br>CAAGAATTTCTGAATTGTTAAAATAA

Table S3: Oligonucleotide primers used in this study.

Primer	Description	Used to construct	Sequence (5'-3')
**DL001**	pUC19-TEF1p-TDH3p	pDL006	TTGTAATTAAAACTTAGATTAGATTGCTATGC
**DL002**	pUC19-TEF1p-TDH3p	pDL006	TTTGTTTGTTTATGTGTGTTTATTCGAAAC
**DL003**	TDH3p-RtMatB	pDL006	CGAATAAACACACATAAACAAACAAAAAAAAAATGTCCAACCACTTGTTCG
**DL004**	RtMatB-CYC1t	pDL006	GGATGAATGCACGCGATTCAGGTTCTAGTGTACAAGTCAGC
**DL010**	SpMae1-ADH1t	pDL006	CGGAATGCGTGCGATTCAAACGGATTCATGTTCAGATGATG
**DL011**	TEF1p-SpMae1	pDL006	AGCAATCTAATCTAAGTTTTAATTACAAAAAAAAATGGGCGAGTTGAAAGAAATC
**DL036**	TDH3p (pair with DL002)	pDL006	CTATGGTGTGTGTCATTATCAATACTGCCATTTCAAAGAATAC
**DL037**	TEF1p (pair with DL001)	pDL006	GTATTGATAATGACACACACCATAGCTTCAAAATGT
**DL076**	pTEF1-BS1_RjCouR-GFP	pDL014	CATTGATGATTGAGAATGTCAATGATTAAGGTATTACAACTTTTTTTACTTCTTGCTCATTAG
**DL077**	p416TEF1-BS1_RjCouR-GFP	pDL014	CCTTAATCATTGACATTCTCAATCATCAATGGAACAAGAAAAATGAAACTGAAACTTG
**DL078**	p416TEF1-BS1_RpCouR-GFP	pDL015	TTGTTATACTCTATAACTATTCTGCACAGTATTACAACTTTTTTTACTTCTTGCTCATTAG
**DL079**	p416TEF1-BS1_RpCouR-GFP	pDL015	CTGTGCAGAATAGTTATAGAGTATAACAAGAACAAGAAAAATGAAACTGAAACTTG
**DL080**	pCCW12-BS2_RjCouR-GFP	pDL016	CATTGATGATTGAGAATGTCAATGATTAAGGTGCTTGATAATCTTTCTTTCCATCC
**DL081**	pCCW12-BS2_RjCouR-GFP	pDL016	CCTTAATCATTGACATTCTCAATCATCAATGTGCCGACTTTATATACTTGAACG
**DL082**	pCCW12-BS2_RpCouR-GFP	pDL017	TTGTTATACTCTATAACTATTCTGCACAGTGCTTGATAATCTTTCTTTCCATCC
**DL083**	pCCW12-BS2_RpCouR-GFP	pDL017	CTGTGCAGAATAGTTATAGAGTATAACAATGCCGACTTTATATACTTGAACG
**DL084**	BS1_RpCouR/BS1_RjCouR	pDL014, pDL015	CTCGTGATACGCCTATTTTTATAGG
**DL085**	BS1_RpCouR	pDL015	AACAATCTAATGAGCAAGAAGTAAAAAAAGTTG
**DL086**	120bp oligo BS23_RpCouR	pDL015	AACTTTTTTTACTTCTTGCTCATTAGATTGTTATACTCTATAACTATTCTGCACAGAAGAAAGCATTGTTATACTCTATAACTATTCTGCACAGTAGCAATCTAATCTAAGTTTTAATTA
**DL087**	120bp oligo BS23_RpCouR	pDL015	TAATTAAAACTTAGATTAGATTGCTACTGTGCAGAATAGTTATAGAGTATAACAATGCTTTCTTCTGTGCAGAATAGTTATAGAGTATAACAATCTAATGAGCAAGAAGTAAAAAAAGTT
**DL088**	p416-TEF1-GFP Backbone	pDL015	GCACAGTAGCAATCTAATCTAAGTTTTAATTACAAAATGC
**DL089**	p416-TEF1-GFP Backbone	pDL014, pDL015	CCTATAAAAATAGGCGTATCACGAG
**DL090**	BS1_RjCouR (pair with DL084)	pDL014	ATCATCAATGTCTAATGAGCAAGAAGTAAAAAAAGTTG

**DL091**	120bp oligo BS23_RjCouR	pDL014	TTTACTTCTTGCTCATTAGACATTGATGATTGAGAATGTCAATGATTAAGGAAGAAAGCACATTGATGATTGAGAATGTCAATGATTAAGGTAGCAATCTAATCTAAGTTTTAATTACAA
**DL092**	120bp oligo BS23_RjCouR	pDL014	TTGTAATTAAAACTTAGATTAGATTGCTACCTTAATCATTGACATTCTCAATCATCAATGTGCTTTCTTCCTTAATCATTGACATTCTCAATCATCAATGTCTAATGAGCAAGAAGTAAA
**DL093**	p416-TEF1-GFP Backbone (pair with DL089)	pDL014	TAAGGTAGCAATCTAATCTAAGTTTTAATTACAAAATGC
**DL110**	NLS_Rj/RpCouR	pDL030, pDL031, pDL032, pDL033	TCATAAGAAATTCGCTCAAACCTTTCTCTTCTTCTTTG
**DL113**	tADH1 - backbone	pDL030, pDL031, pDL032, pDL033	GGCTTTAATTTGCGGCCGGTACGAGCGACCTCATGCTAT
**DL114**	tADH1 - NLS_Rj/RpCouR	pDL030, pDL031, pDL032, pDL033	AAGAGAAAGGTTTGAGCGAATTTCTTATGATTTATGATTTTT
**DL144**	TEF1p	pDL038	ACCAACGGAATGCGTGCGATCAAAATGTTTCTACTCCTTTTTTACTC
**DL145**	TEF1p	pDL038	CATTTTTTTTTTGTAATTAAAACTTAGATTAGATTGCTATG
**DL146**	At4CL1	pDL038	GCAATCTAATCTAAGTTTTAATTACAAAAAAAAAATGGCTCCACAAGAACAAG
**DL147**	At4CL1	pDL038	AGCGGATGAATGCACGCGATTCACAAACCGTTAGCCAAC
**DL168**	RjCouR - ADH2p (pair with DL110)	pDL030, pDL032	ATATCGTAATACACAAAAAAAATGGCTGAATCTCAAG
**DL169**	RpCouR - ADH2p (pair with DL110)	pDL031, pDL033	CTATATCGTAATACACAAAAAAAATGACCTCCTCTAACAG
**DL170**	ADH2p - RjCouR (pair with DL172)	pDL030, pDL032	TTCAGCCATTTTTTTTGTGTATTACGATATAGTTAATAGTTGA
**DL171**	ADH2p - RpCouR (pair with DL172)	pDL031, pDL033	GGAGGTCATTTTTTTTGTGTATTACGATATAGTTAATAGTTGA
**DL172**	ADH2p - backbone	pDL030, pDL031, pDL032, pDL033	ACTCACTATAGGGCGAATTGGGTACCTTAACTGATAGTTTGATCAAAGG
**DL283**	CCW12p (pair with MS016)	pMS07, pMS08, pMS09	TATTGATATAGTGTTTAAGCGAATGA
**DL303**	At4CL1 (pair with MS014)	pMS09	ACCAACGGAATGCGTGCGATTCACAAACCGTTAGCCA
**MS001**	pYX212t	pMS01, pMS04	ATTAAGTCCTCAGCGAGCTGCCGTAAACCACTAAATCG
**MS002**	FBA1t	pMS01, pMS03, pMS04	CCTCAGCACTAGTCCTGCAAGTAAGCTACTATGAAAGACTTTAC
**MS003**	TDH2t	pMS02	ACGGAATGCGTGCGATGCGAAAAGCCAATTAGTGT
**MS004**	FBA1t	pMS02	CAATAGTAATGCCTCAGCACTAGAGTAAGCTACTATGAAAGACTTTAC
**MS005**	TDH2t (pair with MS002)	pSM03	AATTAAGTCCTCAGCGAGCTGCGAAAAGCCAATTAGTGT
**MS008**	FapR	pSMS07, pMS08	AACGGAATGCGTGCGATTTATGAATGTTTTGAACGATACATG
**MS009**	FapR (NLS)	pSMS07, pMS08	CTGTCATTCGCTTAAACACTATATCAATAATGCCAAAGAAGAAGAGAAAG
**MS010**	CCW12p(BS-RpCouR) (pair with DL283)	pMS07	CAGACAGAGCACTAAACTGAACTAGCGACACGCAAAAGAAAAC

**MS011**	CCW12p(BS-RpCouR) (pair with DL283)	pMS08	GCCCCTTTGATCAAACTATCAGTTAAGCGACACGCAAAAGAAAAC
**MS012**	ADH2p	pMS08	CTTAACTGATAGTTTGATCAAAGGG
**MS013**	RpCouR(NLS)	pMS08	CGGATGAATGCACGCGATTCAAACCTTTCTCTTCTTCTTTG
**MS014**	At4CL1 (pair with DL303)	pMS09	CTGTCATTCGCTTAAACACTATATCAATAATGGCTCCACAAGAACAA
**MS016**	CCW12p (pair with DL283)	pMS09	GCCTCAGCACTAGTCCTGCACGACACGCAAAAGAAAAC

Table S4: Oligonucleotide primers used for construction of naringenin strains QL11, NAG1-3, NAG10 and NAG3-1.

Primer	Name	Sequence (5'-3')
**Primers for integration cassettes:** ***XII-4: (TDH3p-At4CL-ADH1t) + (TDH2t-RsCHS-CCW12p)+(tHXT7p-PsCHI-FBAt)***		
P001	*XII-4 up-F*	GTATCCGGCTGTTCCTTCATAG
P002	*XII-4 up-R (with TDH3p-F)*[a]	CTTTGAAATGGCAGTATTGATAATGATAAACTCGATGCCATAGTATGTGTGATGG
P045	*TDH3p-F*	TCGAGTTTATCATTATCAATACTGCC
P046	*TDH3p-R*	CATTTTGTTTGTTTATGTGTGTTTATTCGA
P164	*At4CL-F (with TDH3p-R)*	ACTTAGTTTCGAATAAACACACATAAACAAACAAAATGGCTCCACAAGAACAAG
P165	*At4CL-R (with ADHt-F)*	TTAATAATAAAAATCATAAATCATAAGAAATTCGCTCACAAACCGTTAGCCAAC
P047	*ADH1t-F*	GCGAATTTCTTATGATTTATGATTTTT
P048	*ADH1t-R*	GCATATCTACAATTGGGTGAAATGG
P049	*TDH2t-F*	ATTTAACTCCTTAAGTTACTTTAATGATTTAG
P050	*TDH2t-R (with ADH1t-R)*	AAATCGCTCCCCATTTCACCCAATTGTAGATATGCGCGAAAAGCCAATTAGTGTGATAC
P166	*RsCHS-F (with CCW12p-R)*	CTGTCATTCGCTTAAACACTATATCAATAAACAAAATGGTTACTGTTGAAGATGTTAG
P167	*RsCHS-R (with TDH2t-F)*	AAACTAAATCATTAAAGTAACTTAAGGAGTTAAATTTAAGTACACAATGAATGCAAAAC
P051	*CCW12p-F*	CCACCCATGAACCACACGG
P052	*CCW12p-R*	CATTTTGTTTATTGATATAGTGTTTAAGCGAATG
P053	*tHXT7p-F (with CCW12p-F)*	GCCCCTTTTGGACTAACCGTGTGGTTCATGGGTGGCTCGTAGGAACAATTTCGGG
P054	*tHXT7p-R*	CATTTTTTGATTAAAATTAAAAAAACTTTTTG
P168	*PsCHI-F (with tHXT7p-R)*	CAAAAACAAAAAGTTTTTTTAATTTTAATCAAAAAATGGCAAAACCACCATCTG
P169	*PsCHI-R (with FBAt-F)*	TCATTAAAAAACTATATCAATTAATTTGAATTAACTTATTTTAACAATTCAGAAATTCTTGC
P055	*FBAt-F*	GTTAATTCAAATTAATTGATATAGTTTTTT
P056	*FBAt-R*	GATACCGTCGACCTCGAGTC
P003	*XII-4 dn-F (with FBAt-R)*	CGAGTTCTTTGTAAAGTCTTTCATAGTAGCTTACTATTCCCCATTAGAGTCAAATAAAAG
P004	*XII-4 dn-R*	TTTCTGCCGTACCTGGATGGTCATTTC
**Primers for integration cassettes:** ***XII-1: (TDH3p-At4CL-ADH1t)***		
P005	*XII-1 up-F*	GTTGAGCTCTGTCCTTCATGG
P006	*XII-1 up-R (with TDH3p-F)*	CTTTGAAATGGCAGTATTGATAATGATAAACTCGAGAAAGAACCGAACCGATGCC
P045	*TDH3p-F*	
P046	*TDH3p-R*	
P164	*At4CL-F (with TDH3p-R)*	
P165	*At4CL-R (with ADHt-F)*	
P047	*ADH1t-F*	
P048	*ADH1t-R*	

P007	*XII-1 dn-F (with ADH1t-R)*	AAATCGCTCCCCATTTCACCCAATTGTAGATATGCCTTCCCGTG AATCAACTGCAC
P008	*XII-1 dn-R*	CAATCCTCGCATTTCAGCTTC
**Primers for integration cassettes: *XII-5: (CYC1t-At4CL-TPIp)***		
P009	*XII-5 up-F*	GTAGTGATCATTGGCTTAAC
P010	*XII-5 up-R (with CYCt-R)*	GGACGCTCGAAGGCTTTAATTTGCGGCCGGTACCCGTGACAA TAAATTCAAACCGGT
P057	*CYC1t-F*	GATACCGTCGACCTCGAGTC
P058	*CYC1t-R*	GGGTACCGGCCGCAAATTAA
P170	*At4CL-F (with TPIp-R)*	TCTATAACTACAAAAAACACATACATAAACTAAAAATGGCTCCA CAAGAACAAG
P171	*At4CL-R (with CYCt-F)*	CATAACTAATTACATGACTCGAGGTCGACGGTATCTCACAAAC CGTTAGCCAAC
P059	*TPIp-F*	CATTTTTAGTTTATGTATGTGTTTTTTGTAG
P060	*TPIp-R*	GTTTAAAGATTACGGATATTTAACTTAC
P011	*XII-5 dn-F (with TPIp-F)*	ATTCTAAGTAAGTTAAATATCCGTAATCTTTAAACCAACTCAGAA GTTTGACAGC
P012	*XII-5 dn-R*	CTCTTTTGCCTTTCAAAAAAG
**Primers for integration cassettes: *XII-5: (pYX212t-PsCHI-PGKp)+ (TEF1p-RsCHS-FBAt)***		
P009	*XII-5 up-F*	
P013	*XII-5 up-R (with pYX212t-R)*	GCTCCCTTTAGGGTTCCGATTTAGTGGTTTACGGCGTGACAAT AAATTCAAACCGGT
P061	*pYX212t-F*	TAGGGCCCACAAGCTTACG
P062	*pYX212t-R*	GCCGTAAACCACTAAATCGGA
P172	*PsCHI-F (with PGKp-R)*	AGTAATTATCTACTTTTTACAACAAATATAACAAAATGGCAAAAC CACCATCTG
P173	*PsCHI-R (with pYX212t-F)*	GATACCCGGGTCGACGCGTAAGCTTGTGGGCCCTATTATTTTA ACAATTCAGAAATTCTTGC
P063	*PGKp-F*	ACGCACAGATATTATAACATC
P064	*PGKp-R*	TTTGTTATATTTGTTGTAAAAAGTAGATAA
P065	*TEFp-F (with PGKp-F)*	ATGCCTATTGTGCAGATGTTATAATATCTGTGCGTATAGCTTCA AAATGTTTCTAC
P066	*TEFp-R*	CATTTTGTAATTAAAACTTAGATTAGATTGC
P174	*RsCHS-F (with TEFp-R)*	AAGCATAGCAATCTAATCTAAGTTTTAATTACAAAATGGTTACT GTTGAAGATGTTAG
P175	*RsCHS-R (with FBAt-F)*	TCATTAAAAAACTATATCAATTAATTTGAATTAACTTAAGTACAC AATGAATGCAAAAC
P055	*FBAt-F*	
P056	*FBAt-R*	
P014	*XII-5 dn-F (with FBAt-R)*	CGAGTTCTTTGTAAAGTCTTTCATAGTAGCTTACTCAACTCAGA AGTTTGACAGC
P012	*XII-5 dn-R*	
**Primers for integration cassettes: *XII-1: (pYX212t-PsCHI-PGKp)+ (TEF1p-RsCHS-FBAt)***		
P005	*XII-1 up-F*	

P015	*XII-1 up-R (with pYX212t-R)*	GCTCCCTTTAGGGTTCCGATTTAGTGGTTTACGGCGAAAGAAC CGAACCGATGCC
P061	*pYX212t-F*	
P062	*pYX212t-R*	
P172	*PsCHI-F (with PGKp-R)*	
P173	*PsCHI-R (with pYX212t-F)*	
P063	*PGKp-F*	
P064	*PGKp-R*	
P065	*TEFp-F (with PGKp-F)*	
P066	*TEFp-R*	
P174	*RsCHS-F (with TEFp-R)*	
P175	*RsCHS-R (with FBAt-F)*	
P055	*FBAt-F*	
P056	*FBAt-R*	
P016	*XII-1 dn-F (with FBAt-R)*	CGAGTTCTTTGTAAAGTCTTTCATAGTAGCTTACTCTTCCCGTG AATCAACTGCAC
P008	*XII-1 dn-R*	

[a] short overlaps are indicated in parentheses.

[b] Blue are repeated primers

Table S5: Plasmids used in this study.

Plasmid ID	Description		Origin
**pDL006**	pCfB3036-*SpMae1-RlMatB*	XI-1: (*TEF1p-SpMae1-ADH1t*)+(*TDH3p-RtMatB-CYC1t*)	This study
**pDL014**	p416-*TEF1p*_BS123(*RjCouR*)-*GFP-CYC1t*	CEN.ARS ampR *URA3*	This study
**pDL015**	p416-*TEF1p*_BS123(*RpCouR*)-*GFP-CYC1t*	CEN.ARS ampR *URA3*	This study
**pDL016**	p416-*CCW12p*_BS2(*RjCouR*)-*GFP-CYC1t*	CEN.ARS ampR *URA3*	This study
**pDL017**	p416-*CCW12p*_BS2(*RpCouR*)-*GFP-CYC1t*	CEN.ARS ampR *URA3*	This study
**pDL030**	p416-(*CCW12p*_BS2(*RjCouR*)-*GFP-CYC1t*)+(*ADH2p-RjCouR-CYC1t*)	CEN.ARS ampR *URA3*	This study
**pDL031**	p416-(*CCW12p*_BS2(*RpCouR*)-*GFP-CYC1t*)+(*ADH2p-RpCouR-CYC1t*)	CEN.ARS ampR *URA3*	This study
**pDL032**	p416-(*TEF1p*_BS123(*RjCouR*)-*GFP-CYC1t*)+(*ADH2p-RjCouR-CYC1t*)	CEN.ARS ampR *URA3*	This study
**pDL033**	p416-(*TEF1p*_BS123(*RpCouR*)-*GFP-CYC1t*)+(*ADH2p-RpCouR-CYC1t*)	CEN.ARS ampR *URA3*	This study
**pDL038**	pCfB2909-(*TEF1p-At4CL1-CYC1t*)	XII-5: (*TEF1p-At4CL1-CYC1t*)	This study
**pDL056**	pCfB3042-*KlURA*	2µm ampR *KlURA3* gRNA-X-4	This study
**pDL057**	pCfB3043-*KlURA*	2µm ampR *KlURA3* gRNA-XI-1	This study
**pDL060**	pCfB3050-*KlURA*	2µm ampR *KlURA3* gRNA-XII-5	This study
**pDL074**	pCfB3047-*KlURA*	2µm ampR *KlURA3* gRNA-XII-1	This study
**pDL103**	p416-*CCW12p-GFP-CYC1t*	CEN.ARS ampR *URA3*	This study
**pDL120**	pCfB3053-*KlURA*	2µm ampR *KlURA3* gRNA-XI-1	This study
**pMS01**	pCfB2899-*CHS-CHI*	X-2: (*TEF1p-RsCHS-FBA1t*)+(*PGK1p-PsCHI-pYX212t*)	This study
**pMS02**	pCfB3037-*CHS-CHI*	XI-5: (*CCW12p-RsCHS-TDH2t*)+(*tHXT7p-PsCHI-FBA1t*)	This study
**pMS03**	pCfB3040-*CHS-CHI*	XII-4: (*CCW12p-RsCHS-TDH2t*)+(*tHXT7p-PsCHI-FBA1t*)	This study
**pMS04**	pCfB3035-*CHS-CHI*	X-4: (*TEF1p-RsCHS-FBA1t*)+(*PGK1p-PsCHI-pYX212t*)	This study
**pMS07**	pCfB3038-*CCW12p*_BS2(*RpCouR*)-*FapR*	XII-1: (*CCW12p*_BS2(*RpCouR*)-*FapR- ADH1t*)	This study
**pMS08**	pCfB3038-(*CCW12p*_BS2(*RpCouR*)-*FapR*)-(*ADH2p-RpCouR*)	XII-1: (*CCW12p*_BS2(*RpCouR*)-*FapR-ADH1t*)+(*ADH2p-RpCouR-CYC1t*)	This study
**pMS09**	pCfB2909-(*CCW12p*_BS2(*FapR*)_*At4CL1*)	XII-5: (*CCW12p*_BS2(*FapR*)-*At4CL1-ADH1t*)	This study
**p416TEF**	empty plasmid	CEN.ARS ampR *URA3*	Ref[1]
**p416TEF-GFP**	p416-*TEF1p-GFP-CYC1t*	CEN.ARS ampR *URA3*	Ref[2]
**pFDA09**	p413-(*TEF1p*_NLS_*FapR*) + (*TEF1p*_BS123(*FapR*)-*GFP*	CEN.ARS ampR *HIS3*	Ref[2]
**pX&Y19**	p416-*CCW12p*_BS2(*FapR*)-*GFP-CYC1t*	CEN.ARS ampR *URA3*	Ref[3]
**pCfB2312**	*TEF1p-Cas9-CYC1t*	CEN.ARS ampR *KanMX*	Ref[4]

**Integration cassette backbone plasmids**		
**pCfB2899**	backbone plasmid for integration at X-2	Ref[4]
**pCfB2909**	backbone plasmid for integration at XII-5	Ref[4]
**pCfB3035**	backbone plasmid for integration at X-4	Ref[4]
**pCfB3036**	backbone plasmid for integration at XI-1	Ref[4]
**pCfB3037**	backbone plasmid for integration at XI-5	Ref[4]
**pCfB3038**	backbone plasmid for integration at XII-1	Ref[4]
**pCfB3040**	backbone plasmid for integration at XII-4	Ref[4]

gRNA plasmids			
**pCfB3042**	gRNA plasmid for integration at X-4	2μm ampR *natMX6* gRNA-X-4	Ref[4]
**pCfB3043**	gRNA plasmid for integration at XI-1	2μm ampR *natMX6* gRNA-XI-1	Ref[4]
**pCfB3047**	gRNA plasmid for integration at XII-1	2μm ampR *natMX6* gRNA-XII-1	Ref[4]
**pCfB3050**	gRNA plasmid for integration at XII-5	2μm ampR *natMX6* gRNA-XII-5	Ref[4]
**pCfB3053**	gRNA plasmid for integration at X-2, XI-5, XII-4	2μm ampR *natMX6* gRNA X-2, XI-5, XII-4	Ref[4]
**pQC010**	gRNA plasmid for integration at XII-4	2 μm ampR *KlURA3* gRNA-XII-4.Y	Ref[5]
**pQC032**	gRNA plasmid for integration at XII-1	2μm ampR *URA3* gRNA-XII-1.Y [2x]	Ref[5]
**pQC032**	gRNA plasmid for integration at XII-5	2μm ampR *URA3* gRNA-XII-5.Y [2x]	Ref[5]

Gene templates			
**pDLX_6A3_SpMae1**	template for *SpMae1*		This study
**pDLX_6A3_RtMatB**	template for *RtMatB*		This study
**pDLX_6A3_RjCouR**	template for *RjCouR*		This study
**pDLX_6A3_RpCouR**	template for *RpCouR*		This study
**pG-RsCHS**	template for *RsCHS*		This study
**pG-PsCHI**	template for *PsCHI*		This study
**pCfB854**	template for *At4CL*		Ref[6]

Table S6: Strains used in this study.

Strain ID	Relevant genotype	Parental strain	Origin
**CEN.PK113-11C**	*MAT*a *MAL2-8C SUC2 ura3-52 his3Δ*		
**YFlav06**	*MAT*a *MAL2-8C SUC2 ura3-52 his3Δ XI-1::(ADH1t-SpMae1-TEF1p)+(TDH3p-RtMatB-CYC1t)*	CEN.PK113-11C	This study
**YFlav13**	*MAT*a *MAL2-8C SUC2 ura3-52 his3Δ XII-5::(TEF1p-At4CL1-CYC1t)*	CEN.PK113-11C	This study
**yMS01**	*MAT*a *MAL2-8C SUC2 ura3-52 his3Δ XI-1::(ADH1t-SpMae1-TEF1p)+(TDH3p-RtMatB-CYC1t) X-2::(TEF1p-RsCHS-FBA1t)+(PGK1p-PsCHI-pYX212t), XI-5::(CCW12p-RsCHS-TDH2t)+(tHXT7p-PsCHI-FBA1t), XII-4::(CCW12p-RsCHS-TDH2t)+(tHXT7p-PsCHI-FBA1t)*	yFlav06	
**yMS02**	*MAT*a *MAL2-8C SUC2 ura3-52 his3Δ XI-1::(ADH1t-SpMae1-TEF1p)+(TDH3p-RtMatB-CYC1t) X-2::(TEF1p-RsCHS-FBA1t)+(PGK1p-PsCHI-pYX212t), XI-5::(CCW12p-RsCHS-TDH2t)+(tHXT7p-PsCHI-FBA1t), XII-4::(CCW12p-RsCHS-TDH2t)+(tHXT7p-PsCHI-FBA1t) X-4::(TEF1p-RsCHS-FBA1t)+(PGK1p-PsCHI-pYX212t)*	yMS01	
**yMS04**	*MAT*a *MAL2-8C SUC2 ura3-52 his3Δ XI-1::(ADH1t-SpMae1-TEF1p)+(TDH3p-RtMatB-CYC1t) X-2::(pYX212t-PsCHI-PGK1p)+(TEF1p-RsCHS-FBA1t) XI-5::(TDH2t-RsCHS-CCW12p)+(tHXT7p-PsCHI-FBA1t), XII-4::(TDH2t-RsCHS-CCW12p)+(tHXT7p-PsCHI-FBA1t) X-4::(pYX212t-PsCHI-PGK1p)+(TEF1p-RsCHS-FBA1t) XII-5::(ADH1t-At4CL1-CCW12p_BS2_FapR)*	yMS02	This study
**yMS05**	*MAT*a *MAL2-8C SUC2 ura3-52 his3Δ XI-1::(ADH1t-SpMae1-TEF1p)+(TDH3p-RtMatB-CYC1t) X-2::(pYX212t-PsCHI-PGK1p)+(TEF1p-RsCHS-FBA1t) XI-5::(TDH2t-RsCHS-CCW12p)+(tHXT7p-PsCHI-FBA1t), XII-4::(TDH2t-RsCHS-CCW12p)+(tHXT7p-PsCHI-FBA1t) X-4::(pYX212t-PsCHI-PGK1p)+(TEF1p-RsCHS-FBA1t) XII-5::(ADH1t-At4CL1-CCW12p_BS2_FapR) XII-1::(ADH1t-FapR-CCW12p_BS2_RpCouR)*	yMS04	This study
**yMS06**	*MAT*a *MAL2-8C SUC2 ura3-52 his3Δ XI-1::(ADH1t-SpMae1-TEF1p)+(TDH3p-RtMatB-CYC1t) X-2::(pYX212t-PsCHI-PGK1p)+(TEF1p-RsCHS-FBA1t) XI-5::(TDH2t-RsCHS-CCW12p)+(tHXT7p-PsCHI-FBA1t), XII-4::(TDH2t-RsCHS-CCW12p)+(tHXT7p-PsCHI-FBA1t) X-4::(pYX212t-PsCHI-PGK1p)+(TEF1p-RsCHS-FBA1t) XII-5::(ADH1t-At4CL1-CCW12p_BS2_FapR) XII-1::(ADH1t-FapR-CCW12p_BS2_RpCouR)+(ADH2p-RpCouR-CYC1t)*	yMS04	This study
**QL11**	*MAT*a *ura3-52* can1Δ::*cas9*-*natNT2 TRP1 LEU2 HIS3 XII-2::(GPM1p-AtPAL2-FBA1t)+(TDH3p-AtC4H-CYC1t)+(tHXT7p-AtATR2-pYX212t)+(PGK1p-CYB5-ADH1t) X-3::(TPI1p-EcaroL-pYX212t)+(ADH1t-ARO7 G141S-TEF1p)+(PGK1p-ARO4 K229L-CYC1t) X-4::(CYC1t-ARO1-TPI1p)+(TDH3p-ARO2-ADH1t)+(TDH2t-ARO3-TEF1p)*		Ref[5]
**NAG10**	*MAT*a *ura3-52* can1Δ::*cas9*-*natNT2 TRP1 LEU2 HIS3 XII-2::(GPM1p-AtPAL2-FBA1t)+(TDH3p-AtC4H-CYC1t)+(tHXT7p-AtATR2-pYX212t)+(PGK1p-CYB5-ADH1t) X-3::(TPI1p-EcaroL-pYX212t)+(ADH1t-ARO7 G141S-TEF1p)+(PGK1p-ARO4 K229L-CYC1t) X-4::(CYC1t-ARO1-TPI1p)+(TDH3p-ARO2-ADH1t)+(TDH2t-ARO3-TEF1p) XII-4: (TDH3p-At4CL-ADH1t) + (TDH2t-RsCHS-CCW12p)+(tHXT7p-PsCHI-FBAt)*		This study
**NAG1-3**	*MAT*a *ura3-52* can1Δ::*cas9*-*natNT2 TRP1 LEU2 HIS3 XII-2::(GPM1p-AtPAL2-FBA1t)+(TDH3p-AtC4H-CYC1t)+(tHXT7p-AtATR2-pYX212t)+(PGK1p-CYB5-ADH1t) X-3::(TPI1p-EcaroL-pYX212t)+(ADH1t-ARO7 G141S-TEF1p)+(PGK1p-ARO4 K229L-CYC1t) X-4::(CYC1t-ARO1-TPI1p)+(TDH3p-ARO2-ADH1t)+(TDH2t-ARO3-TEF1p) XII-4: (TDH3p-At4CL-ADH1t) + (TDH2t-RsCHS-CCW12p)+(tHXT7p-PsCHI-FBAt) XII-1:(pYX212t-PsCHI-PGKp)+ (TEF1p-RsCHS-FBAt) XII-5: (pYX212t-PsCHI-PGKp)+ (TEF1p-RsCHS-FBAt)*		This study
**NAG3-1**	*MAT*a *ura3-52* can1Δ::*cas9*-*natNT2 TRP1 LEU2 HIS3 XII-2::(GPM1p-AtPAL2-FBA1t)+(TDH3p-AtC4H-CYC1t)+(tHXT7p-AtATR2-pYX212t)+(PGK1p-CYB5-ADH1t) X-3::(TPI1p-EcaroL-pYX212t)+(ADH1t-ARO7 G141S-TEF1p)+(PGK1p-ARO4 K229L-CYC1t) X-4::(CYC1t-ARO1-TPI1p)+(TDH3p-ARO2-ADH1t)+(TDH2t-ARO3-TEF1p) XII-4: (TDH3p-At4CL-ADH1t) + (TDH2t-RsCHS-CCW12p)+(tHXT7p-PsCHI-FBAt) XII-1: (TDH3p-At4CL-ADH1t) XII-5: (CYC1t-At4CL-TPIp)*		This study

References

(1) Shen, H. J.; Cheng, B. Y.; Zhang, Y. M.; Tang, L.; Li, Z.; Bu, Y. F.; Li, X. R.; Tian, G. Q.; Liu, J. Z. Dynamic control of the mevalonate pathway expression for improved zeaxanthin production in *Escherichia coli* and comparative proteome analysis. *Metab. Eng.* **2016**, *38*, 180-190. DOI: 10.1016/j.ymben.2016.07.012.

(2) David, F.; Nielsen, J.; Siewers, V. Flux control at the malonyl-CoA node through hierarchical dynamic pathway regulation in *Saccharomyces cerevisiae*. *ACS Synth. Biol.* **2016**, *5* (3), 224-233. DOI: 10.1021/acssynbio.5b00161.

(3) Dabirian, Y.; Li, X.; Chen, Y.; David, F.; Nielsen, J.; Siewers, V. Expanding the dynamic range of a transcription factor-based biosensor in *Saccharomyces cerevisiae*. *ACS Synth. Biol.* **2019**, *8* (9), 1968-1975. DOI: 10.1021/acssynbio.9b00144.

(4) Jessop-Fabre, M. M.; Jakočiūnas, T.; Stovicek, V.; Dai, Z.; Jensen, M. K.; Keasling, J. D.; Borodina, I. EasyClone-MarkerFree: A vector toolkit for marker-less integration of genes into *Saccharomyces cerevisiae* via CRISPR-Cas9. *Biotechnol. J.* **2016**, *11* (8), 1110-1117. DOI: 10.1002/biot.201600147.

(5) Liu, Q.; Yu, T.; Li, X.; Chen, Y.; Campbell, K.; Nielsen, J.; Chen, Y. Rewiring carbon metabolism in yeast for high level production of aromatic chemicals. *Nat. Commun.* **2019**, *10* (1), 4976. DOI: 10.1038/s41467-019-12961-5.

(6) Li, M.; Kildegaard, K. R.; Chen, Y.; Rodriguez, A.; Borodina, I.; Nielsen, J. De novo production of resveratrol from glucose or ethanol by engineered *Saccharomyces cerevisiae*. *Metab. Eng.* **2015**, *32*, 1-11. DOI: 10.1016/j.ymben.2015.08.007.
